# A new styracosternan hadrosauroid (Dinosauria: Ornithischia) from the Early Cretaceous of Portell, Spain

**DOI:** 10.1371/journal.pone.0253599

**Published:** 2021-07-07

**Authors:** Andrés Santos-Cubedo, Carlos de Santisteban, Begoña Poza, Sergi Meseguer

**Affiliations:** 1 Àrea de Cristal·lografia i Mineralogia, Departament de Ciències Agràries i del Medi Natural, Universitat Jaume I, Castelló, España; 2 Grup Guix, Vila-real, Castelló, España; 3 Departament de Botànica i Geologia, Universitat de València, Burjassot, València, España; State Museum of Natural History, GERMANY

## Abstract

A new styracosternan ornithopod genus and species is described based on the right dentary of a single specimen from the Mirambell Formation (Early Cretaceous, early Barremian) at the locality of Portell, (Castellón, Spain). *Portellsaurus sosbaynati* gen. et sp. nov. is diagnosed by two autapomorphic features as well as a unique combination of characters. The autapomorphies include: the absence of a bulge along the ventral margin directly ventral to the base of the coronoid process and the presence of a deep oval cavity on the medial surface of the mandibular adductor fossa below the eleventh-twelfth tooth position. Phylogenetic analyses reveal that the new Iberian form is more closely related to the African taxon *Ouranosaurus nigeriensis* than to its synchronic Iberian taxa *Magnamanus soriaensis* and *Iguanodon galvensis*. In addition, *Portellsaurus sosbaynati* is less related to other Iberian taxa such as *Iguanodon bernissartensis* and *Proa valdearinnoensis* than to the other Early Cretaceous Iberian styracosternans *Mantellisaurus atherfieldensis* and *Morelladon beltrani*. A new phylogenetic hypothesis is proposed that resolves *Iguanodon* (*I*. *bernissartensis*, *I*. *galvensis*) with the Valanginian *Barilium dawsoni* into a monophyletic clade (Iguanodontoidea). The recognition of *Portellsaurus sosbaynati* gen. et sp. nov. as the first styracosternan dinosaur species identified from the Margas de Mirambell Formation (early Barremian–early late Barremian) in the Morella sub-basin (Maestrat Basin, eastern Spain) indicates that the Iberian Peninsula was home to a highly diverse assemblage of medium-to-large bodied styracosternan hadrosauriforms during the Early Cretaceous.

## Introduction

Of particular importance to the understanding of the evolution of Early Cretaceous ornithopods is the analysis of species of hadrosauroids phylogenetically nested between *Iguanodon* and Hadrosauromorpha. Most of these taxa are known from the Early Cretaceous of Europe and Asia, although a few are known from the Late Cretaceous. Previous studies have produced a series of conflicting or poorly resolved phylogenetic hypotheses of the relationships within Hadrosauroidea [1–6].

The Early Cretaceous European fossil record of styracosternan hadrosauriforms is divided into iguanodontoids: *Barilium*–*Iguanodon* and hadrosauroids: *Hypselospinus* + more derived taxa [7]. Currently, the Lower Cretaceous Iberian styracosternan iguanodontoids species are the lower Barremian *Iguanodon galvensis* [8] and the upper Barremian *Iguanodon bernissartensis* [9]. On the other hand, Hadrosauroids are the upper Barremian *Morelladon beltrani* [5] and *Mantellisaurus atherfieldensis* [10], and the lower Albian *Proa valdearinnoensis* [11]. Norman [12] considered the lower Barremian ‘*Delapparentia turolensis*’ [13] a potential *nomen dubium*.

Recent discoveries such as the fossil from Portell (Spain) described below might shed new light on the knowledge of hadrosauroid evolution.

## Abbreviations

Institutional abbreviations: MQ-II, Mas de Curolles II site, Portell, Spain; NHMUK, The Natural History Museum, London, UK; MAP, Museo Aragonés de Paleontología, Teruel, Spain; CMP-MS, Mas de la Parreta Quarry-Mas de Sabater, Morella, Spain; RBINS, Royal Belgian Institute of Natural Sciences, Brussels, Belgium; AR, Santa María de Ariño Mine, Teruel, Spain; MNHN, Musée National d’Histoire Naturelle, Paris, France; YHZ, Yizhou Fossil Museum, Yixian, China.

## Geographical and geological context

**Fig 1:**
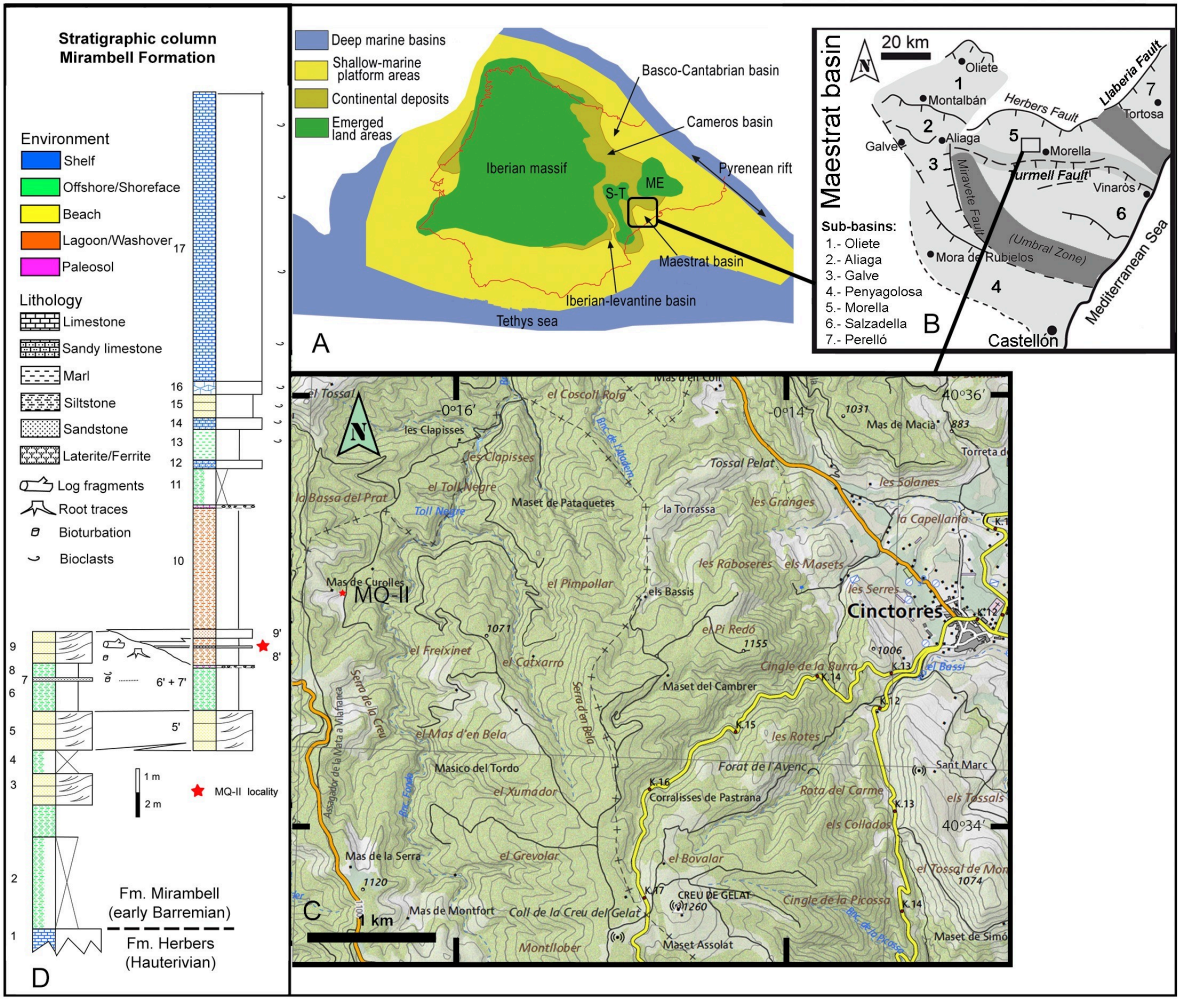
Geographical and geological location of the MQ-II fossil locality in Portell (Castellón, Spain). (A) Palaeogeographic basins of the Iberian Plate during Early Cretaceous, modified from Santos-Cubedo et al. [14] under a CC BY license, with permission from Loisele Ediciones, original copyright [2016]. (B) Palaeogeographic sub-basins within the Maestrat Basin and active faults during Early Cretaceous sedimentation, modified from Fig 1A in Holgado et al. [15] under a CC BY license 4.0. (C) Geographical location of the MQ-II site. Base map from CV50 2019 CC BY 4.0 © Institut Cartogràfic Valencià, Generalitat. (D) Stratigraphic column for the Mirambell Formation in the MQ-II locality.

Portell is located 110 km north of Castelló de la Plana, in eastern Spain (Fig 1). The municipality of Portell is in the Maestrat Basin in the Iberian Range. The Mas de Curolles-II (MQ-II) site was discovered in 1998 as the result of a geological prospecting campaign. The outcrops studied are located around the villages of Portell, La Mata and Cinctorres (Castellón province) and La Cuba (Teruel province).

Geologically, the site belongs to the Mirambell Formation and, palaeogeographically, this area belongs to the north-western margin of the Morella sub-basin, within the Maestrat Basin (eastern part of the Iberian Chain, Fig 1) [15].

From bottom to top, it encompasses the Early Cretaceous Wealden facies (Fig 1): the lacustrine Herbers Formation, the litoral-lacustrine Mirambell Formation, the shallow marine Artoles Formation, and the transitional Morella Formation [14, 16]. The ages of the Early Cretaceous continental units were determined by studying the charophyte assemblages from the local series [17] and from ammonoid findings and numerical ages derived from 87Sr/86Sr values measured on rudist, oyster and brachiopod shells [18].

In the case of the Mirambell Formation under study, the age is early Barremian to early late Barremian, corresponding to the *Atopochara trivolvis triquetra biozone* [16, 19].

The Mirambell Formation here has a thickness of 19 m (Fig 1) and the unit is formed by an alternation of successive limestones, marls, carbonates and sandstones that evolve from continental environments to a shallow marine platform [17].

## Materials and methods

### Paleontological ethics statements

The specimen of *Portellsaurus sosbaynati* gen. et sp. nov. described in this paper (catalog number MQ98-II-1) was discovered by Miquel Guardiola, Julián Yuste and Silvia Fabregat in 1998, previous to the law “4/1998, de 11 de junio, del Patrimonio Cultural Valenciano”, that regulates field works in the Valencian Community. Later, the fossil was deposited in the Colección Museográfica de Cinctorres (Castellón, Spain). This collection was recognized by Valencian government as a public museum collection by resolution of 23 April of 1999.

MQ98-II-1 is accessioned in the Palaeontology collections of the Colección Museográfica de Cinctorres (Castellón, Spain) and was examined, photographed, and measured there, with the written permission of the director of the institution.

### Nomenclatural acts

The electronic edition of this article conforms to the requirements of the amended International Code of Zoological Nomenclature, and hence the new names contained herein are available under that Code from the electronic edition of this article. This published work and the nomenclatural acts it contains have been registered in ZooBank, the online registration system for the ICZN. The ZooBank LSIDs (Life Science Identifiers) can be resolved and the associated information viewed through any standard web browser by appending the LSID to the prefix "http://zoobank.org/". The LSID for this publication is: urn:lsid:zoobank.org:pub:7F1B7DFC-A4BD-4B36-AC55-7130DFA056E7. The electronic edition of this work was published in a journal with an ISSN, and has been archived and is available from the following digital repositories: PubMed Central, LOCKSS.

## Results

### Systematic palaeontology

Dinosauria Owen, 1842 [20]
Ornithischia Seeley, 1887 [21]
Ornithopoda Marsh, 1881 [22]
Iguanodontia Dollo, 1888 [23] *sensu* Sereno, 2005 [24]
Ankylopollexia Sereno, 1986 [25] *sensu* Sereno, 2005 [24]
Styracosterna Sereno, 1986 [25] *sensu* Sereno, 2005 [24]
Hadrosauriformes Sereno, 1997 [26] *sensu* Sereno, 1998 [27]
Hadrosauroidea Cope, 1870 [28] *sensu* Sereno, 2005 [24]
*Portellsaurus* gen nov.
urn:lsid:zoobank.org:act:3E240477-3C7C-4BDD-A450-60FBC74AFEE9
*Portellsaurus sosbaynati* sp nov.
urn:lsid:zoobank.org:act:60FC0FE9-95DF-4D43-8A40-41FEFDE2DDBF

#### Etymology

The generic designation combines the name of *Portell*, the town in which the fossil was discovered, with the Greek *sauros* (lizard). The specific name is taken from Vicente Sos Baynat, a Spanish geologist born in Castelló de la Plana and the first scientist to be honoured with the title of “Doctor Honoris Causa” by Universitat Jaume I.

#### Holotype

MQ98-II-1, almost complete right dentary, stored at the Colección Museográfica de Cinctorres (Castellón, Spain).

#### Locality and horizon

A site near Mas de Curolles, Portell, Castellón (Spain). The beds exposed belong to the Margas de Mirambell Formation, which is early Barremian to early late Barremian (~130–129 Ma) [18]. MQ98-II-1 was recovered in a sandstone layer between two siltstone strata that were deposited on a lagoon/washover environment (Fig 1). Universal Transverse Mercator (UTM) coordinates of the Mas de Curolles-II (MQ-II) site are: 30T 730302 4496335.

#### Diagnosis of genus and species by monotypy

Styracosternan hadrosauroid distinguished by two autapomorphies: the absence of a bulge along the ventral margin directly ventral to the base of the coronoid process, convergent with the two more derived hadrosauroids *Altirhinus kurzanovi* [29] and *Sirindhorna khoratensis* [30], and the presence of a deep oval cavity on the medial surface of the mandibular adductor fossa below the eleventh-twelfth tooth position. Internally, the cavity connects with the last nutrient foramina (the most caudal).

It is also diagnosed by the following unique combination of features: (1) a straight morphology of the ventral margin of the rostral ramus (convergent with *Iguanodon* [7, 37], *Ouranosaurus* [31], *Barilium* [36], *Lanzhousaurus* [33], *Jinzhousaurus* [37], *Bolong* [3], and *Dakotadon* [34]); (2) rostral tip not raised respect to the ventral margin (unlike in *Ouranosaurus* [31], and *Bolong* [3]); (3) dorsal and ventral margins parallels (unlike in *I*. *galvensis* [7], and *O*. *nigeriensis* [31] with divergent dorsal and ventral margins); (4) presence of a diastema (unlike in *Lanzhousaurus* [33], and *Dakotadon* [34]); (5) absence of a bulge along ventral margin directly ventral to the base of the coronoid process (unlike in *Jinzhousaurus* [37], and *Ouranosaurus* [31]).

### Description and comparison of MQ98-II-1

Measurements of MQ98-II-1 are given in Table 1. Alveoli are numbered from rostral to caudal; thus, the most rostral alveolus is referred to as the ‘first’ and so on.

**Table 1:** Measurements of MQ98-II-1.

Element	Measurements	mm
Dentary (R)	1. Rostrocaudal length (measured along a straight line).	313
	2. Maximum dorsoventral height of the dentary ramus.	108
	3. Maximum lateral width.	82
	4. Length of preserved tooth row, measured from rostral-most point of first alveolus to caudal-most point of last preserved alveolus (measured as a straight line).	303
	5. Maximum opening of the adductor fossa caudally.	39

Right Dentary dimensions. Measurements are in millimetres.

**Fig 2:**
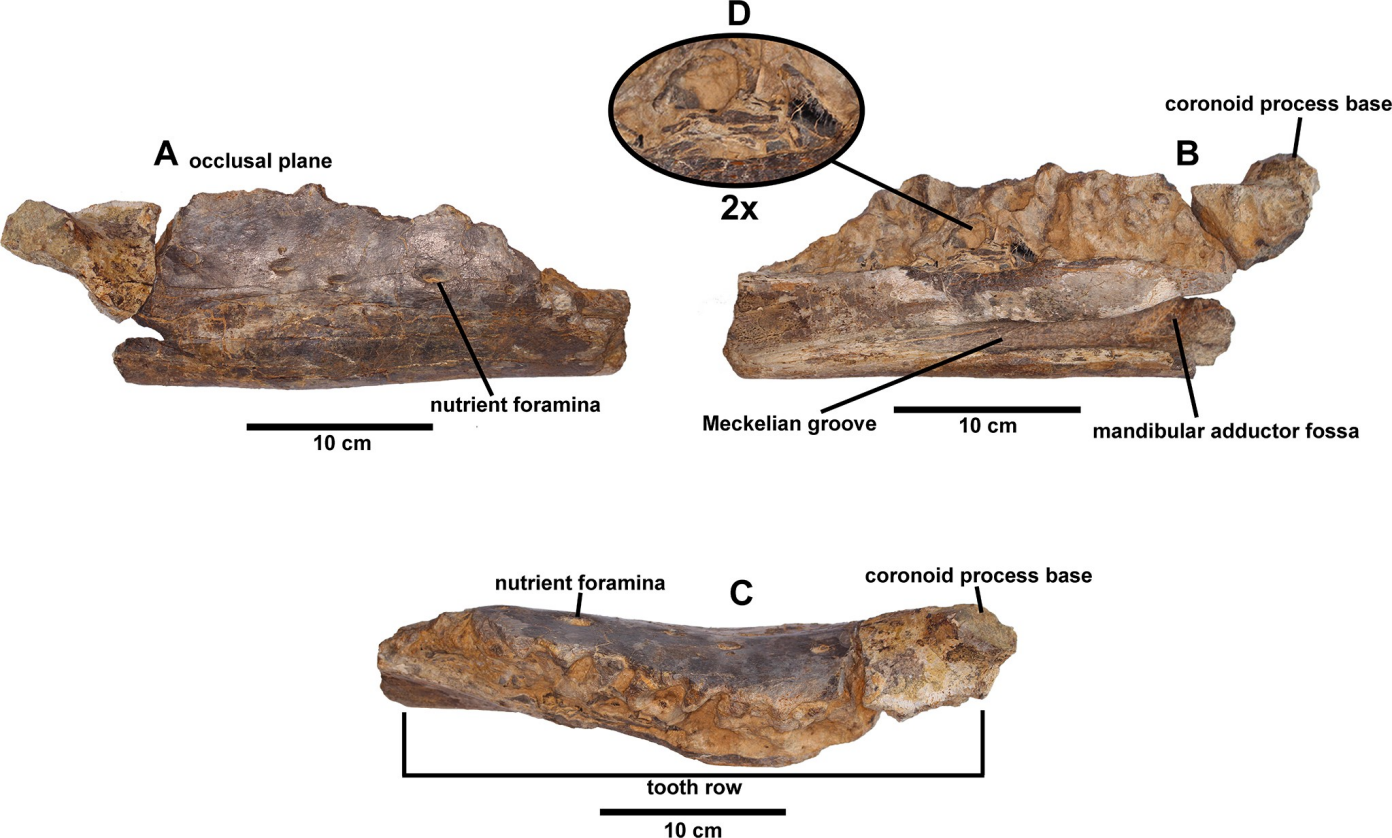
Right dentary (MQ98-II-1) of *Portellsaurus sosbaynati*. Labial (A), lingual (B), and occlusal (C) views. (D) Enlargement (2x) of a dental crown fragment at the tooth row. Scale bar equals 10 cm.

The dentary ramus of the specimen is partially preserved, but that preserves practically all the tooth row. Only the predentary, a little part of the rostral region and the coronoid process are missing. The dentary ramus, in lateral view, is nearly perfectly straight and robust, with parallel dorsal and ventral edges. It is not deflected downwardly, in contrast to *Mantellisaurus* [38]. The tooth row, in medial and lateral view (Fig 2A and 2B), is straight as in other non-hadrosaurid iguanodontians. In *Owenodon* [32], and *Proa* [11] it is moderately convex.

The alveolar row in dorsal view is medially arched from the middle to the caudal part (Fig 2C), the tooth row converges both caudally and rostrally in relation to the lateral surface of the teeth, resembling the condition in other styracosternans. This row seems to terminate slightly caudal to the rostral end, so the jaw would have a diastema. A diastema is absent in *Proa* [11], *Lanzhousaurus* [33], *Owenodon* [32], *Dakotadon* [34] and *Fukuisaurus* [35].

Several plesiomorphic characters can be observed in the tooth row of MQ98-II-1, including tooth-shaped alveoli not forming parallel vertical walls (Fig 2A), a single replacement tooth per tooth family and probably one tooth per tooth position in the occlusal plane, as in *Owenodon* [32], *Barilium* [36], *Fukuisaurus* [35], *Lanzhousaurus* [33], *Iguanodon* [37], *Mantellisaurus* [38], *Hypselospinus* [12], and *Ouranosaurus* [31]. The dentary preserves fifteen alveoli and practically all of the teeth have been lost from their respective alveoli. Parts of the crown of three teeth are found in the fifth, sixth and seventh tooth alveoli positions (Fig 2A). These fragments can be seen in alveolar hollows that show the edges of the teeth with marginal denticles typical of the ankylopollexians (Fig 2D). The teeth have ridges on the lingual surface of the crown and they have a main crest that is arranged distally. Moreover, the marginal denticles are tongue-shaped with mameloned edges.

Although the major part of the coronoid process is missing, the most caudal tooth undoubtedly extended medial to the coronoid process but still rostral to the longitudinal axis of the process (Fig 2C), as in *Ouranosaurus* [31], *Owenodon* [32], and *Dakotadon* [34]. There is no broad shelf separating the coronoid process and the tooth row (Fig 2C), which resembles the condition in *Fukuisaurus* [35], *Barilium* [36], *Iguanodon* [37], *Mantellisaurus* [38], *Ouranosaurus* [31], *Bolong* [39], *Hypselospinus* [12], *Jinzhousaurus* [40], *Owenodon* [37], *Hippodraco* [41], and *Dakotadon* [34].

Just below the coronoid process, the ventral margin is smooth in MQ98-II-1 (Fig 2B). There is no ventral bulge in contrast to the condition found in *Mantellisaurus* [38], *Ouranosaurus* [31], *Hypselospinus* [12], *Jinzhousaurus* [40], *Probactrosaurus* [42], and *Equijubus* [43]. However, a lateral bulge gives rise to the coronoid process, as in *Iguanodon* [37], *Mantellisaurus* [38], *Ouranosaurus* [31], *Proa* [11], *Altirhinus* [29], *Jinzhousaurus* [40], and *Equijubus* [43]. The base of the coronoid process is caudally inclined, as in *Ouranosaurus* [31], *Hypselospinus* [12], *Lanzhousaurus* [33], *Owenodon* [37], *Dakotadon* [34], and *Bolong* [39].

**Fig 3:**
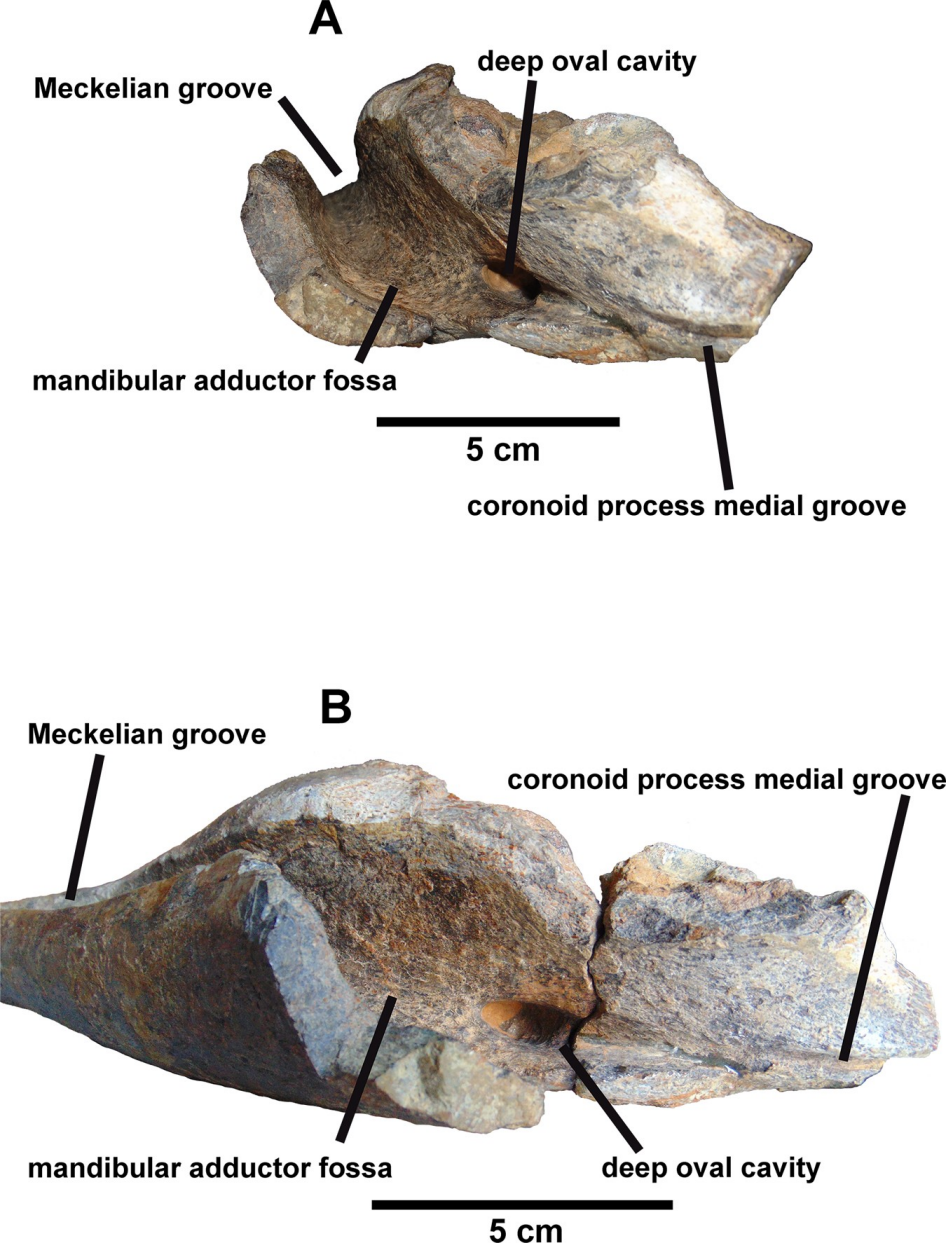
Caudal part of the right dentary (MQ98-II-1). In Caudal (A) and caudo-ventral (B) views. Scale bar equals 5 cm.

Five large oval neurovascular foramina pierce the lateral surface of the dentary (Fig 2A). In medial view a well-developed Meckelian groove progressively broadens caudally, forming the rostral part of the adductor fossa (Fig 2B). The inner edge of the coronoid process is separated from the outer edge by a channel that opens ventrally in the adductor fossa, as in *Ouranosaurus* [31]. We call this channel the coronoid process medial groove (Fig 3A) and it becomes a deep oval cavity on the medial surface of the mandibular adductor fossa below the eleventh-twelfth tooth position (Fig 3B). This cavity has an oval section that measures 21 mm long and 9 millimeters wide. Regarding the depth, it is 33 mm deep, although the end of it cannot be seen since it is partially filled with sediment. Internally, the hole connects with the last neurovascular foramen (most caudal) of the external part of the mandible (Fig 2A) and its presence causes a bulging on the lateral surface of the dentary, that gives rise to the coronoid process (this bulge is also present in *Iguanodon* [37], *Mantellisaurus* [38], *Ouranosaurus* [31], *Proa* [11], *Altirhinus* [29], *Jinzhousaurus* [40], and *Equijubus* [43]).

The presence of this cavity is not reported in other hadrosauriforms, but there are few detailed descriptions of this region of the dentary in these dinosaurs. We believe that this is a diagnostic character of this jaw and not a pathology as will be developed in the Discussion section.

**Fig 4:**
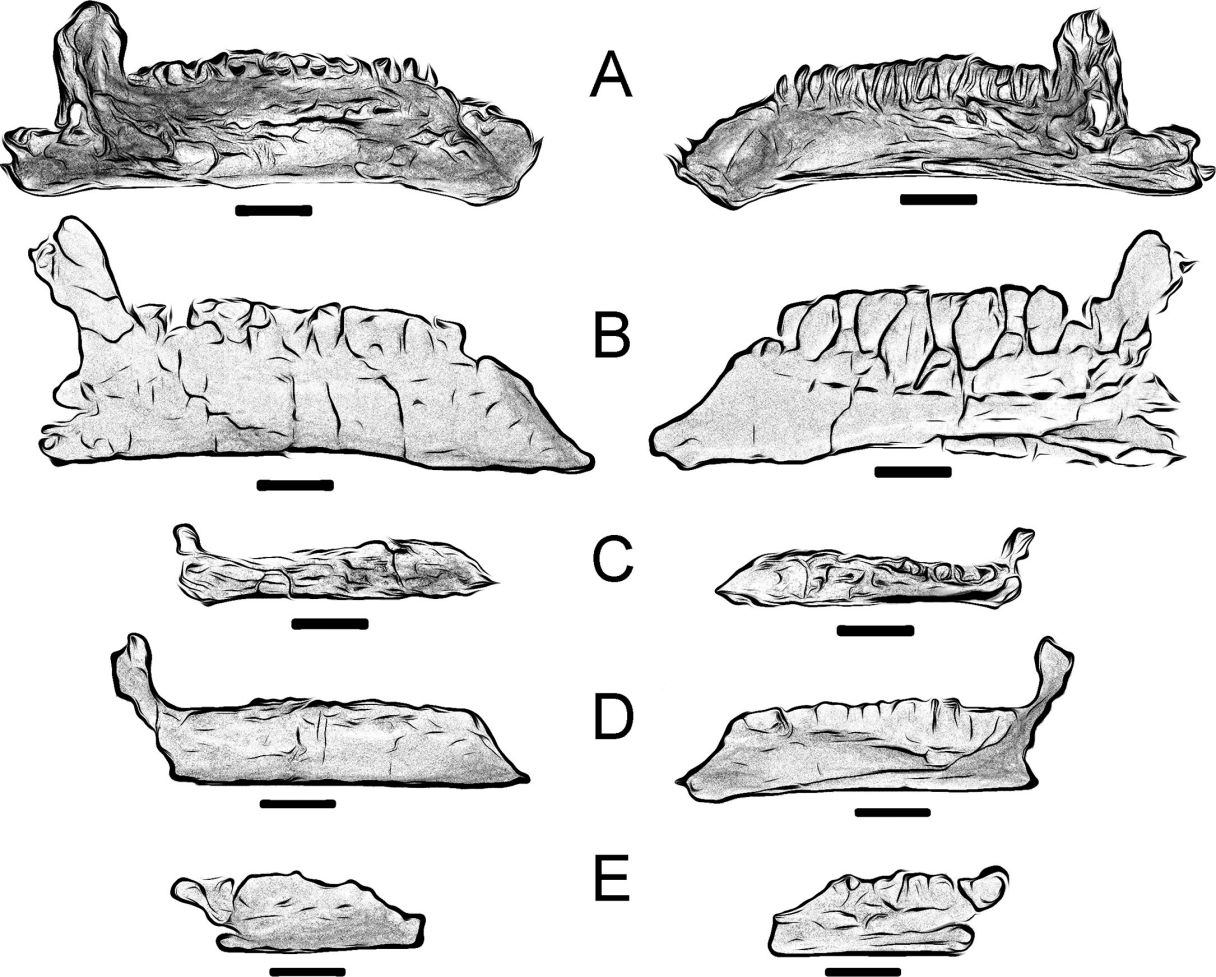
Straight ventral margin of the rostral ramus of some styracosternans from the Lower Cretaceous in comparison with *P*. *sosbaynati* gen. et sp. nov. Medial and lateral views. Scale bar equals 10 cm. (A) *Iguanodon bernissartensis* (IRSNB-1561, vertically reversed). (B) *Lanzhousaurus magnidens* (GSLTZP01-001, vertically reversed, drawing from Fig 1 in You, Ji and Li [33]). (C) *Ouranosaurus nigeriensis* (MNHN GDF 300, vertically reversed, drawing from Fig 29 in Taquet [31]). (D) *Barilium dawsoni* (NHMUK PV R 28660, drawing from Fig 2 in McDonald, Barrett and Chapman [36]). (E) *Portellsaurus sosbaynati* gen. et sp. nov. (MQ98-II-1).

**Fig 5:**
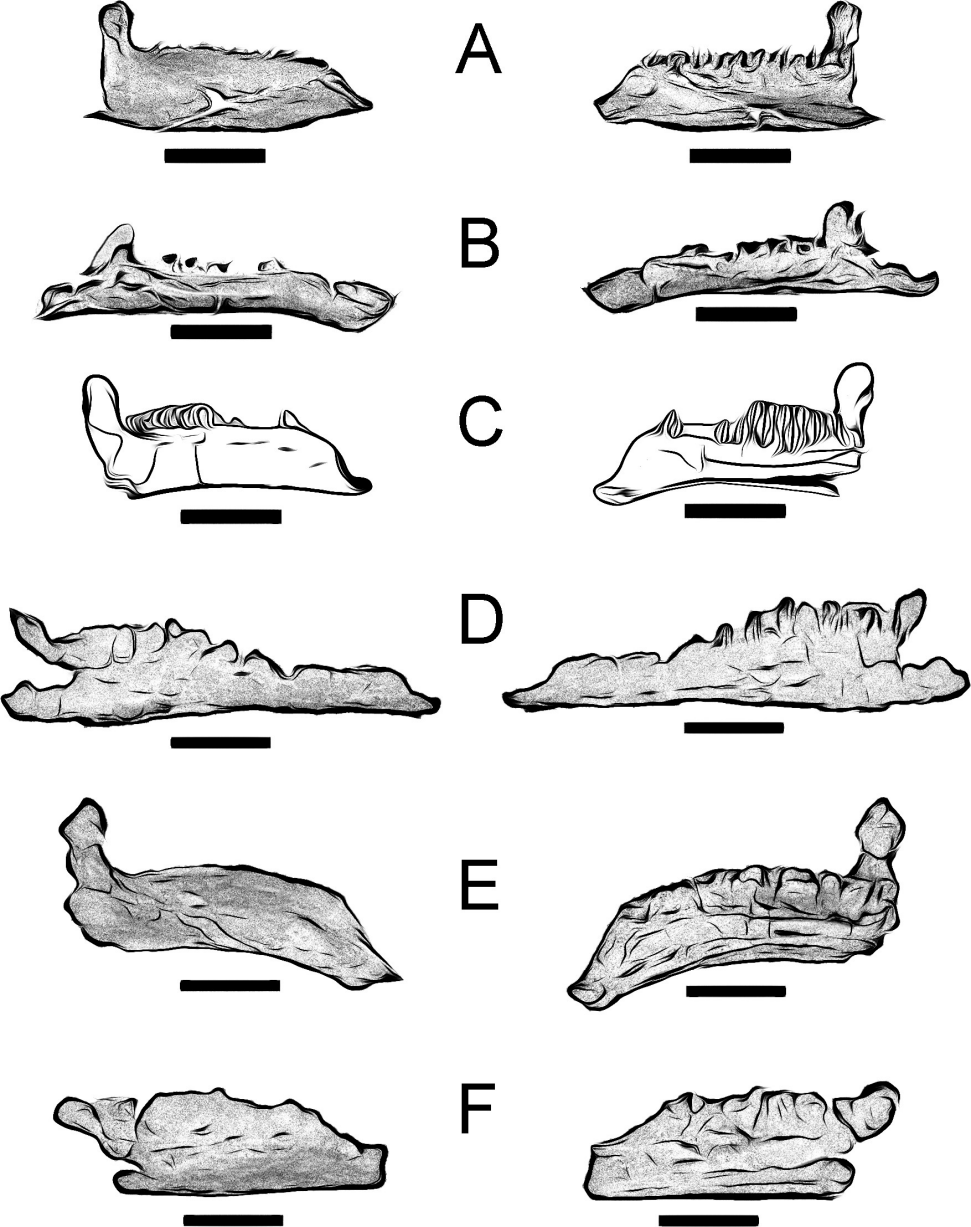
Inflected ventral margin of the rostral ramus of some styracosternans from the Lower Cretaceous in comparison with *P*. *sosbaynati* gen. et sp. nov. Medial and lateral views. Scale bar equals 10 cm. (A) *Fukuisaurus tetoriensis* (FPDM-V-40-9, vertically reversed, drawing from Fig 4 in Kobayashi and Azuma [35]). (B) *Mantellisaurus atherfieldensi*s (NHMUK PV R 11521, vertically reversed, drawing from Fig 3 in McDonald [54]). (C) *Probactrosaurus gobiensis* (PIN 2232/42-1, drawing from Fig 12 in Rozhdestvensky [42]). (D) *Hypselospinus fittoni* (NHMUK PV R 1831, drawing from Fig 36 in Norman [12]). (E) *Proa valdearinnoensis* (AR-1-1365, drawing from Fig 6 in McDonald et al. [11]). (F) *Portellsaurus sosbaynati* gen. et sp. nov. (MQ98-II-1).

Finally, it is important to note that McDonald et al. [36, 41] considered the morphology of the ventral margin of the rostral ramus leading to the predentary articulation a taxonomically decisive character, since they have not observed intraspecific variations in the morphology of the rostral branch in their studies. In this case, *Portellsaurus sosbaynati* gen. et sp. nov. has a straight morphology of the ventral margin of the rostral ramus, as in *Iguanodon* [7, 37], *Ouranosaur*us [31], *Barilium* [36], *Lanzhousaurus* [33], *Jinzhousaurus* [37], *Bolong* [3], and *Dakotadon* [34] (Fig 4). Conversely, it curves gently towards the predentary articulation and symphysis (it is inflected ventrally) in *Mantellisaurus* [38], *Proa* [11], *Altirhinus* [29], *Hypselospinus* [12], *Probactrosaurus* [42], *Equijubus* [43], and *Hippodraco* [41] (Fig 5). Furthermore, the margin curves dorsally towards the symphysis in *Fukuisaurus* [35] (Fig 5A).

If McDonald et al. [36, 41] are right and morphology of the ventral margin of the rostral ramus is a taxonomically decisive character, *Portellsaurus sosbaynati* gen. et sp. nov. must be close to *Iguanodon* [7, 37], *Ouranosaurus* [31], *Barilium* [36], *Lanzhousaurus* [33], *Jinzhousaurus* [37], *Bolong* [3], and *Dakotadon* [34]. But MQ98-II-1 differs from aforementioned taxa in these characteristics:

- Presence of a dentary diastema (unlike in *Dakotadon* [34], and *Lanzhousaurus* [33]).
- Tooth row extents medial to coronoid process but still rostral to longitudinal axis of the process (unlike in *I*. *galvensis* [7], *I*. *bernissartensis* [37], *Barilium* [36], and *Lanzhousaurus* [33]).
- Dentary dorsal and ventral margins are parallels (unlike in *I*. *galvensis* [7], and *Ouranosaurus* [31]).
- Absence of a bulge along ventral margin directly ventral to the base of the coronoid process (unlike in *Ouranosaurus* [31], and *Jinzhousaurus* [37]).
- Absence of a dentary platform between the tooth row and the coronoid process (unlike in *Lanzhousaurus* [33]).
- Coronoid process caudally inclined (unlike in *I*. *bernissartensis* [37], *Barilium* [36], and *Jinzhousaurus* [37]).
- Dentary rostral tip not raised respect to the ventral margin (unlike in *I*. *galvensis* [7], *Ouranosaurus* [31], and *Bolong* [3]).

## Discussion

Morphological features present in other styracosternan dentaries are explained in order to better understand the similarities and differences between them and the remains recovered from Mas de Curolles II.

### Comparison with other styracosternans from the Lower Cretaceous of Europe

#### Valanginian

Valanginian large styracosternans comprises *Barilium dawsoni* [44] and *Hypselospinus fittoni* [12] from England. Other taxa from this age are considered nomina dubia or subjective synonyms of *Barilium* or *Hypselospinus* [45, 46].

*Barilium dawsoni* (Lydekker, 1888) [47]

Holotype: NHMUK PV R 798, R 798b, R 799, R 800–R 806, R 4771, R 4742. Despite the variety of registered numbers, all refer to a single partial skeleton collected from one quarry in the village of Shornden, near Hastings (East Sussex), United Kingdom. It was found in the Wadhurst Clay Formation (Valanginian).

Sites: In the Lower Cretaceous of Europe, specifically in England.

The following description is based on text and figures from McDonald et al. [36].

Dentary of *B*. *dawsoni* (NHMUK PV OR 28660) is straight and robust as in MQ98-II-1, but the latter is short in comparision with the very large dentary of *B*. *dawsoni*. In lateral view, it has parallel dorsal and ventral margins, so the rostral ramus is straight, as in MQ98-II-1. Rostral to the first tooth alveolus, the dorsal margin of the dentary deepens ventrally. At this point the rostral ramus curves medially towards the symphysis. The symphysis is horizontal. It is directed rostrolaterally to caudomedially respect to the lateral surface of the dentary. On the rostral margin of the dentary, lateral to the symphysis, can be observed a deep groove for reception of the lateral process of the predentary. On the ventral surface of the symphyseal region there is a shallow depression. This forms the articulation surface for the ventromedial process of the predentary.

A short diastema is present. On the medial surface of the dentary, caudal to the symphysis, begins the Meckelian groove. It extends caudally. This channel gradually expands along its length, as in MQ98-II-1. Caudally it becomes a broad, shallow depression that forms the rostral portion of the adductor fossa, like in Portell specimen.

There is a vertical coronoid process, with an expanded rostral margin and a straight and unexpanded caudal margin in lateral view. At mid-height, it reaches its maximum thickness, despite it flattens towards its apex. The medial surface of the coronoid process is divided into two portions by a shelf. This shelf arises at the base of the coronoid process and curves dorsally along the medial surface, dividing the medial surface into a smooth rostrodorsal portion and a rougher caudoventral ones.

There are a series of neurovascular foramina. Eight small foramina are present from the rostral tip to the predentary groove. This series continues as a row of eight larger foramina that pierce the dorsolateral surface of the dentary. Four small foramina are located from the ventral surface of the symphyseal region onto the lateral surface of the dentary, and two large elliptical foramina are caudally situated to this row of foramina, on the ventrolateral surface. Only five large oval neurovascular foramina are present in MQ98-II-1, and there are located more ventral respect to the occlusal plane than in *B*. *dawsoni* specimen.

Only eighteen U-shaped tooth alveoli are preserved. The tooth row is straight and begins to curve caudolaterally at the fourteenth alveolus, in contrast to MQ98-II-1 that begins to curve caudolaterally at the tenth alveolus. So, it is bowed medially in dorsal view.

In summary, *Barilium dawsoni* [44] differs from Portell dentary in: the absence of a bulge on the lateral surface ventral to the coronoid process that gives rise to the process; the absence of a deep oval cavity on the medial surface of the mandibular adductor fossa below the eleventh-twelfth tooth position; the position of the caudal-most extent of tooth row with respect of the longitudinal axis of the coronoid process; the inclination of that process and the number of neurovascular foramina.

*Hypselospinus fittoni* (Lydekker, 1889) [48]

Holotype: NHMUK PV R 1635. Fragmentary skeleton found in Wadhurst Clay Formation (Valanginian) in Hastings (East Sussex), United Kingdom.

Sites: In the Lower Cretaceous of Europe, specifically in England.

The following description is based on text and figures from Norman [12].

This dentary is fractured and crushed. Despite this, the outline of the upper margin of the dentary is deep and robust, in addition to being reasonably straight, as in MQ98-II-1. The dentary symphysis is horizontal, in medial view. Near to the symphysis, the ventral surface of the dentary is shallowly arched ventrally, in contrast to MQ98-II-1. There is a short and smooth projection beyond and lateral to the symphyseal surface. There is a modest diastema. Only the upper portion of the coronoid process is preserved, and it is positioned laterally and adjacent to the posterior alveoli. All of the functional dentition has also been broken away and many of the replacement crowns, are lost. Anteriorly, there is a Meckelian groove that extends caudally. It becomes a broad adductor fossa posteriorly.

In summary, *Hypselospinus fittoni* [12] differs from Portell dentary in: the absence of a bulge along the ventral margin directly ventral to the base of the coronoid process; the absence of a deep oval cavity on the medial surface of the mandibular adductor fossa below the eleventh-twelfth tooth position; the absence of a bulge on the lateral surface ventral to the coronoid process that gives rise to the process; the morphology of the ventral margin of the rostral ramus and the position of the caudal-most extent of tooth row with respect of the longitudinal axis of the coronoid process.

#### Late Hauterivian–early Barremian

Another styracosternan, *Magnamanus soriaensis*, a medium-sized quadrupedal dinosaur (9–10 m long) was described from the late Hauterivian–early Barremian in Spain [49].

*Magnamanus soriaensis* Fuentes Vidarte, Meijide Calvo, Meijide Fuentes and Meijide Fuentes, 2016 [49].

Holotype: 2000/132, 2001/122, 2002/95, 2003/69 and 2004/54. This is a partial skeleton including a fragmentary left dentary found in the Golmayo Formation (late Hauterivian–early Barremian) in Golmayo (Soria), Spain.

Sites: In the Lower Cretaceous of Europe, specifically in Spain.

The following description is based on text and figures from Fuentes Vidarte et al. [49].

The external surface of the left dentary is smooth and slightly curved. The medial surface, which is practically parallel to the previous one, does not present a dental shelf but is marked by the dental alveoli, giving it a certain wavy appearance. The coronoid process is relatively low. It is tilted back and towards the mesial side of the tooth. The external surface of the process is slightly convex; the medial surface is concave near the apex and convex at its base. The cranial border is rounded and the caudal one is a fine, cutting edge from which some radial striations arise from the caudal zone of the process. Apart from the coronoid process and a little of the upper part of the tooth row, no other elements of the dentary have been preserved that allow a better comparison with MQ98-II-1.

#### Barremian

Barremian styracosternans comprise ‘*Delapparentia turolensis*’ [13], *Iguanodon galvensis* [8], and *Morelladon beltrani* [5] from Spain. Norman [12] considers ‘*Delapparentia*’ a potential nomen dubium. In this case we cannot compare our specimen with *D*. *turolensis* because no dentary have been described.

*Iguanodon galvensis* Verdú, Royo-Torres, Cobos and Alcalá, 2015 [8].

Holotype: MAP-4787, a sub-adult specimen that includes disarticulated cranial and postcranial bones from the Camarillas Formation (early Barremian) Galve (Teruel), Spain.

Sites: In the Lower Cretaceous of Europe, specifically in Spain.

The following description is based on text and figures from Verdú et al. [7].

The tooth row is straight, in medial and lateral view. But, the tooth row converges, in dorsal view, both caudally and rostrally respect to the lateral wall. The tooth row is bowed lingually along its caudal half, in dorsal view. These characters are similar in MQ98-II-1.

Although in lateral view, dorsal and ventral edges of MAP-4787 (dentary holotype) are parallel, and the dentary ramus is robust and straight, this right dentary ramus is partially preserved, and the rostral region is missing [7]. Even if, a small fragment of a juvenile right dentary (MPG-SCH-10) it has been considered by Verdú et al. [7] as a paratype of *Iguanodon galvensis*. The ventral and dorsal edges of the fossil diverge rostrally, developing a deep-rostral end as in *Ouranosaurus* [31], and *Sirindhorna* [30]. Conversely, the ventral and dorsal edges of *Portellsaurus sosbaynati* are parallel.

Associated with this feature, there is an autapomorphic character for *I*. *galvensis* that is absent in all other iguanodontians, the presence of a short, abrupt, and marked convexity over the dorsal edge close to the symphyseal region [7]. This feature is not present in MQ98-II-1.

Parallel to the tooth row, there are three small neurovascular foramina, on the dorsolateral side of the dentary. In this row, the last teeth extended caudal to the longitudinal axis of the coronoid process, but it is still rostral to the caudal margin of this process. In contrast, specimen from Portell presents five large oval neurovascular foramina on the lateral surface of the dentary, and the most caudal tooth extended medial to the coronoid process but still rostral to the longitudinal axis of the process (Fig 2C).

Between the tooth row and the coronoid process no broad shelf is present. Below the coronoid process no ventral bulge is observed. A well-developed lateral bulge gives rise to the coronoid process. These characters are the same in MQ98-II-1.

Sixteen tooth-shaped alveoli are preserved, which correspond to a single replacement tooth per tooth family. In the occlusal plane, there is only one tooth per tooth position.

On the medial surface of the dentary, there is a Meckelian groove. It progressively broadens caudally, that forms the rostral portion of the adductor fossa. The contact with the surangular is irregular and the caudal edge of the dentary is oblique.

In summary, *Iguanodon galvensis* [7, 8] differs from Portell dentary in: the absence of a deep oval cavity on the medial surface of the mandibular adductor fossa below the eleventh-twelfth tooth position; the position of the caudal-most extent of tooth row with respect of the longitudinal axis of the coronoid process; the dentary shape in medial view; the morphology of the rostral tip respect to the ventral margin of the dentary and the number of neurovascular foramina.

*Morelladon beltrani* Gasulla, Escaso, Narváez, Ortega and Sanz, 2015 [5].

Holotype: specimen CMP-MS-03 is a partial skeleton including a complete right dentary tooth found in the Arcillas de Morella Formation (late Barremian [50]) in Morella (Castellón), Spain.

Sites: In the Lower Cretaceous of Europe, specifically in Spain.

The following description is based on text and figures from Gasulla et al. [5].

*Morelladon* is mainly characterized by elongated dorsal neural spines forming a ‘sail’ on its back. The type specimen does not include a dentary, but a right dentary tooth is preserved (CMP-MS-03-89). It preserves the proximal portion of the root and the basal half of the crown. The crown is heavily worn and the marginal denticles on the mesial and distal margins are not preserved. The crown is narrow labiolingually and expanded mesiodistally. The lingual surface of the crown is enamelled and bears a prominent distally offset primary ridge. Two narrow, subparallel accessory ridges are located mesial to the primary ridge. The labial surface of the crown possesses an almost vertical, slightly concave wear facet [5]. We cannot compare MQ98-II-1 with *M*. *beltrani* because no dentary of the latter species has been found.

#### Middle Barremian–early Aptian

*Iguanodon bernissartensis* and *Mantellisaurus atherfieldensis* are known from several sites in Europe and probably coexisted [32]. Other taxa from the middle Barremian–early Aptian are considered nomina dubia or synonyms of *Iguanodon* and *Mantellisaurus* [45, 46].

*Iguanodon bernissartensis* Boulenger in van Beneden, 1881 [51]

The following description is based on text and figures in Norman [32, 37] and first-hand examination of specimens from Belgium (RBINS collection). *I*. *bernissartensis* was a large and robust iguanodontoid (10–13 m in length) from the middle Barremian–early Aptian [7, 52].

Holotype: specimen RBINS R51 (1534) is a complete articulated skeleton [37] found in the Sainte-Barbe Clays Formation (middle Barremian–earliest Aptian [52]) of Bernissart, in Hainault (Belgium).

Sites: In the Lower Cretaceous of Europe, specifically in Germany, Belgium, Spain, France, and the United Kingdom [7, 9, 10, 32, 37, 45, 46].

In the dentaries of *Iguanodon bernissartensis*, described by Norman [37], the rostral end of the dentary tapers towards a rounded projection along its ventral edge, just behind and medially to the horizontal symphyseal suture. The dorsal margin of the dentary curves gently upwards and backwards and, against this surface, joins the predentary; immediately below this margin, there are some large vascular grooves. Behind the predentary suture, the dorsal margin of the dentary becomes horizontally backward as a perfectly scalloped alveolar ridge holding functional teeth. Medially, teeth are retained by a thin alveolar parapet; its surface texture and its vascular supply are identical to those from the maxilla.

Regarding the backward area of the first alveoli, in dorsal view, the dentary increasingly thickens until it reaches the coronoid process. As in all advanced ornithischian dinosaurs, tooth rows are aligned along the medial edge of the dentary, where a platform is developed between the alveolar margin and the lateral wall of the dentary. Functional teeth follow a straight line in the dentary, but when they approach the coronoid process, they are bent out towards their base. In medial view, below the alveolar parapet, there is an elongated slot known as Meckel’s canal. This is a shallow channel posteriorly enlarged and opened in the mandibular fossa (adductor fossa). There is a long and shallow depression in the dentary, just above the Meckelian groove, marking the facet for the prearticular.

The caudal end of the dentary is somewhat complex to allow firm attachment of the remaining mandibular bones. The coronoid process is prominently marked on its medial surface by oblique grooves and ridges whose function is to fix the coronoid bone. The caudal surface of the coronoid process is sharp and overlaps the surangular. Under subsequent alveoli, the medial surface of the dentary forms a thin rearward process that borders the medial mandibular fossa, where the prearticular is supported.

*I*. *galvensis* and *I*. *bernissartensis* have features in common, but other number of osteological differences. Both species have a robust and straight dentary, as is the case of MQ98-II-1. The dentary of *I*. *galvensis* has a dorsal convexity located close to the symphyseal region (autapomorphic character), that is not present in *I*. *bernissartensis* neither in MQ98-II-1.

In summary, *Iguanodon bernissartensis* [37] differs from Portell dentary in: the absence of a deep oval cavity on the medial surface of the mandibular adductor fossa below the eleventh-twelfth tooth position; the position of the caudal-most extent of tooth row with respect of the longitudinal axis of the coronoid process; the inclination of that process and the number of neurovascular foramina.

*Mantellisaurus atherfieldensis* (Hooley, 1925) [53]

The following description is based on text and figures in Hooley [53], Norman [32, 38], and McDonald [54].

Holotype: the specimen NHMUK R5764 is composed of a large skull fragment and a partially articulated skeleton found in Brook Bay, on the Isle of Wight (United Kingdom), within the Wessex Formation (Upper Barremian) [53]. In addition to being recovered on the Isle of Wight, this species was also located in the Sainte-Barbe Clays Formation (Bernissart), middle Barremian–earliest Aptian [52].

Sites: fossils have been found in Belgium, the United Kingdom and Spain [5, 38, 45, 46], although there is also material tentatively referred to this taxon from Spain [10, 55], Germany [56], and France [57].

Norman [38] described this genus based on the individual IRSNB 1551, from Bernissart, and all the material deposited in the Natural History Museum (London) from the Isle of Wight and from Ockley (Surrey). In dentaries of *Mantellisaurus atherfieldensis*, the lower jaw is firmly built despite having a small and weak dental symphysis. However, the symphysis is held by the predentary. The main body of each jaw is curved longitudinally, with teeth medially positioned and with a high laterally situated coronoid process. Behind this process, the jaw drops sharply to the jaw joint. Behind the glenoid, there is a small retroarticular upturned process. The mandibular fossa (adductor fossa) is quite narrow and is confined to the area between the coronoid process and the glenoid cavity. The lower jaw stands out for the proximity of each mandibular ramus leaving a narrow, parallel sides. The dentary tapers to a rounded point anterior and lateral to a small and horizontal symphysis. Dorsal margins curve upwards and backwards marking the suture with the predentary, where there is a short diastema before the development of the scalloped edge of the dental battery. Teeth are not anchored in independent holes, but rather in a continuous groove and are preserved medially by an alveolar parapet. Although there are no interdental plates, inner dental alveolar walls of the dentary are moulded to the shape of the emerging roots and teeth crowns for continuous support. Below the alveolar parapet, there is a shallow longitudinal groove with regularly spaced grooves penetrating into the base of the alveolar groove, presumably supplying nutrients to the dental plates. Laterally to the dentition, there is a wide platform (cavity of the cheek) at the caudal end where the coronoid process starts to grow. Medially, below the alveolar parapet, there is the groove that forms Meckel’s canal, which is long and rostrally shallow, but becomes caudally wider opening into the mandibular fossa.

In summary, *Mantellisaurus atherfieldensis* [38] differs from Portell dentary in: the absence of a bulge along the ventral margin directly ventral to the base of the coronoid process; the absence of a deep oval cavity on the medial surface of the mandibular adductor fossa below the eleventh-twelfth tooth position; the morphology of the ventral margin of the rostral ramus; the position of the caudal-most extent of tooth row with respect of the longitudinal axis of the coronoid process and the inclination of that process.

#### Early Albian

Finally, a single taxon of large styracosternan, *Proa valdearinnoensis* [11] has been described in the early Albian of Spain.

*Proa valdearinnoensis* McDonald, Espílez, Mampel, Kirkland and Alcala, 2012 [11].

Holotype: The specimen AR-1/19 is composed of a partial skeleton found in the Escucha Formation and is composed of a disarticulated but associated skull that includes premaxilla, partial maxilla, quadrate, supraorbitals, braincase and skull roof, predentary, left dentary, partial right surangular (AR-1-2012), right dentary (AR-1-2013), some isolated teeth (AR-1-2014) and several postcranial bones [11].

Site: In the Lower Cretaceous of Spain [11].

The dentary of the specimen AR-1/19, in dorsal view, has a straight tooth row from the first alveoli to the twelfth approximately. From there, the tooth row curves caudolaterally towards the base of the coronoid process. The tooth row is dorsally convex in lateral and medial views, a feature only seen in *Owenodon* (NHMUK R2998). Respect the base of the coronoid process, the tooth row extends caudally. The dorsal and ventral margins of the dentary are parallel. The ventral margin of the rostral ramus is inflected ventrally to the symphysis. The alveoli have the shape of the dentary teeth. The coronoid process is offset laterally from the first tooth row by a narrow shelf. The coronoid process is expanded rostrocaudally along its rostral and caudal margins.

In summary, *Proa valdearinnoensis* [11] differs from Portell dentary in: the absence of a dentary diastema; the absence of a deep oval cavity on the medial surface of the mandibular adductor fossa below the eleventh-twelfth tooth position; the shape of the tooth row in lateral view; the position of the caudal-most extent of tooth row with respect of the longitudinal axis of the coronoid process; the morphology of the ventral margin of the rostral ramus and the inclination of the coronoid process.

### Comparison with other closely related styracosternans (see Phylogenetic analysis)

*Ouranosaurus nigeriensis* Taquet, 1976 [31]

Holotype: The specimen MNHN GDF 300, a mostly complete skeleton recovered from the Elrhaz Formation, upper Aptian of Niger [31].

Site: Several paleontological sites in the Lower Cretaceous of Niger.

According to the original description by Taquet [31], the dentary of *Ouranosaurus nigeriensis* is the largest element of the mandible. It is an elongated bone, whose front part is very high and transversely flattened. The back is less high, thicker and is bent on the medial side. The height of the dentary decreases notably from the front to the back. The rostral end of the dentary is strongly curved medially. The ventral margin of the symphysis is spout-shaped and curves downwards ventrally. The rostral end of the dentary is higher in the mandibular symphysis and the rostral edge of the dentary extends forward horizontally, beyond the first alveoli, measuring 60 mm in length before curving ventrally and medially to the level of the symphysis. This frontal extension of the dorsal edge of the dentary offsets exactly the rostral extension of the premaxilla, which is extended far beyond the anterior end of the maxilla. Consequently, the caudal end of the predentary and the first dentary tooth alveoli move away from each other to remain separated by a diastema.

The rostral margin of the dentary is double and includes an arcuate medial edge that joins it medially at the level of the symphysis, and an outer edge separated from the first by a thin channel. The outer edge is thick and extends slightly beyond the anterior limit of the mandibular symphysis. The outer surface of the dentary presents some grooves below the alveolar ridge. This alignment extends from the coronoid process to the end of the anterior margin of the dentary. The tooth row is external to the axis of the elongation of the dentary, and curves towards the caudal part in a “flattened S-shape”. The dorsolateral edge of the dentary, in front of the coronoid process joins with the anterolateral edge of this process by the elevation of the occlusal surface of the dentary. The alveolar row is hidden in lateral view.

The Meckelian groove extends along the ventral edge of the medial side of the dentary and tapers from the caudal edge of the symphysis to below the coronoid process. This channel is shaped like a concave and somewhat narrow groove. This groove widens towards the back and posteriorly fills most of the height of the bony ventral edge of the dentary at the base of the coronoid process.

The coronoid process of the mandible of *Ouranosaurus nigeriensis* is very solid, and rises above the occlusal surface. It is located in the lateral margin of the dentary, medially to the jugal. This process is inclined backwards, as in most reptiles, while it is almost vertical in *I*. *bernissartensis*. Instead, it is inclined forwards in hadrosaurs. The outer surface of the coronoid process is bent and is continuous with the convex outer surface of the dentary. Its medial surface is slightly concave. The posterior edge of the process is double, with a right outer edge, oblique from the top to the bottom, and from the back to the right, and with a serrated inner edge. The inner edge is separated from the outer edge by a deep channel that opens ventrally in the adductor fossa.

Dental alveoli are coated by a thin bony plate forming an integral part of the dentary (named the alveolar parapet by Hooley [53]). This is poorly preserved and there are only a few fragments of the plate.

In summary, *Ouranosaurus nigeriensis* [31] differs from Portell dentary in: the absence of a deep oval cavity on the medial surface of the mandibular adductor fossa below the eleventh-twelfth tooth position; the shape of the dentary in lateral or medial view; presence of a bulge along ventral margin directly ventral to the base of the coronoid process and the morphology of the rostral tip respect to the ventral margin of the dentary.

*Bolong yixianensis* Wu, Godefroit and Hu, 2010 [39]

Holotype: The specimen YHZ-001, a nearly complete skeleton recovered from the Yixian Formation, Late Barremian–Early Aptian of China [39].

Site: Two sites in the Lower Cretaceous of China [3].

According to the description by Wu and Godefroit [3], left and right dentaries are preserved in the holotype (YHZ-001).

It has subparallel dorsal and ventral margins, so the main body of the dentary is gently dorsoventrally convex along most of its length, that differs from MQ98-II-1 with straight ramus. The rostral ramus is not significantly downturned, as in MQ98-II-1. In this view, the rostral tip is located above the ventral third of the ramus, and the rostrodorsal articular surface for the predentary fills less than two-thirds of the height of the dentary.

There is a diastema which is not larger than two crown widths. The parapet is lower than in other hadrosauroids. This parapet is thin and limited ventrally by a series of interconnected nutritional foramina.

Fourteen tooth positions are present, which differs from the Portell specimen that preserves fifteen alveoli in the dentary.

The coronoid process is inclined slightly caudally to the long axis of the dentary. Laterally, it is offset with respect to the tooth row. Between this row and the coronoid process there is no broad shelf separation.

Anteriorly, there is a Meckelian groove that extends caudally. It becomes a broad adductor fossa posteriorly.

In summary, *Bolong yixianensis* [39] differs from Portell dentary in: the absence of a deep oval cavity on the medial surface of the mandibular adductor fossa below the eleventh-twelfth tooth position and the morphology of the rostral tip respect to the ventral margin of the dentary.

### Deep oval cavity in MQ98-II-1 dentary: An apomorphic character or a paleopathology?

To the present work, it is important to clarify if the cavity existent in the dentary is a character present in the species or it could be a pathological artefact. To try to discern between one possibility or the other, a detailed study of pathologies present in the dinosaur fossil record has been carried out.

First of all, it is important to note that paleopathologist remarks that disease occurrence in dinosaurs is very infrequent [58–61 and references therein]. Recently, Hamm et al. [58] presented a study in which they combine the analysis of CT images and systematic phylogenetic disease bracketing, for diagnosis of pathologies in fossils. The authors conclude that in extinct dinosaurs (non-avian) the most common diseases are fractures and traumas, followed by infections (post-traumatic and non-traumatic). Neoplastic disease is rare in vertebrate fossils [58, 59, 61], also few metabolic disorders have been described, such as Paget’s disease or gout [58]. Other authors, suggested that pathological processes are independent of phylogeny and time [60].

**Fig 6:**
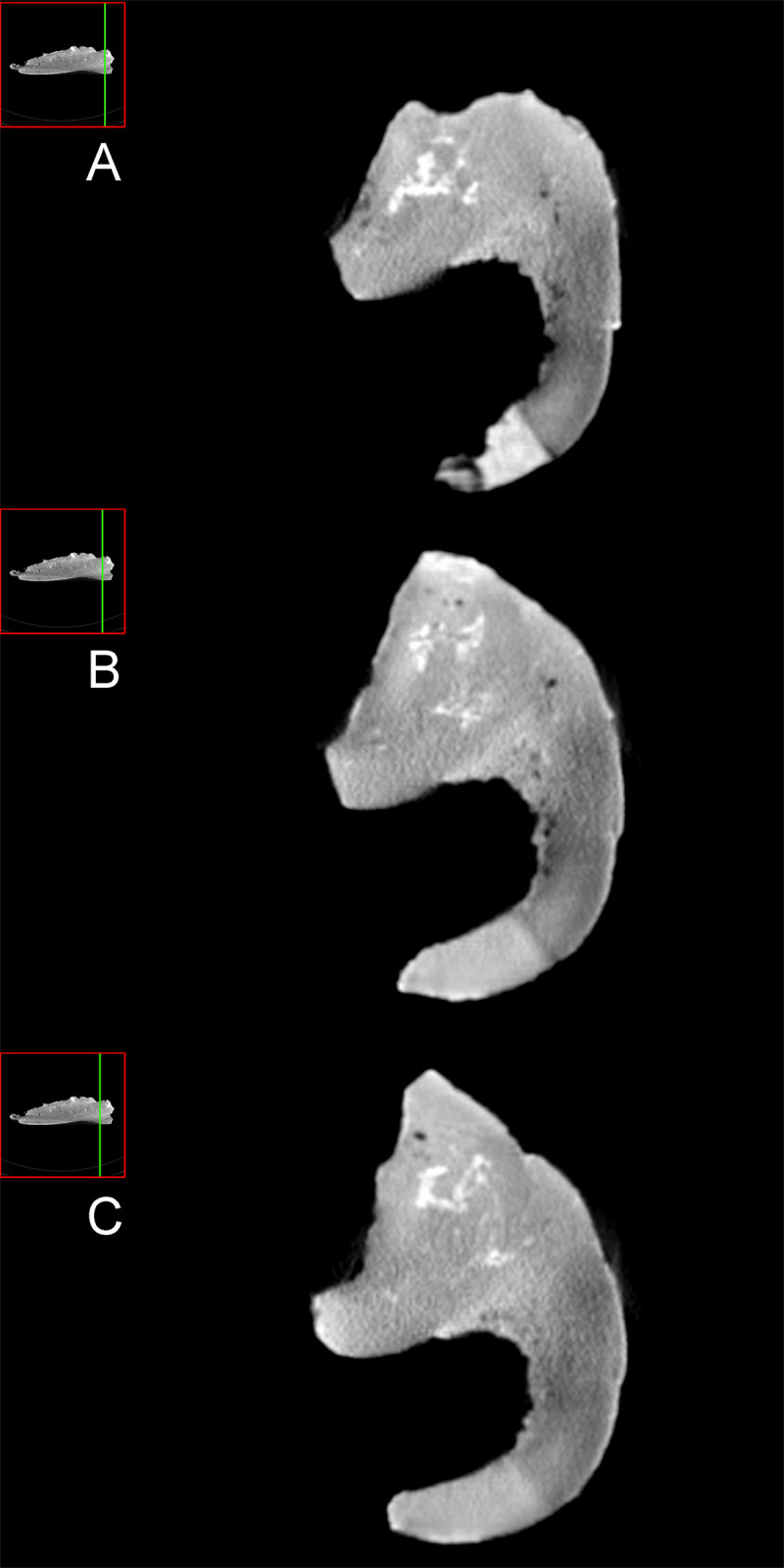
MQ98-II-1 dentary tomographic slices in the axial plane. A) General view illustrating the scan area and CT image of axial slice no. 416. B) General view illustrating the scan area and CT image of axial slice no. 406. C) General view illustrating the scan area and CT image of axial slice no. 397.

For the present study we combine the analysis of CT images and systematic phylogenetic disease bracketing, following Hamm et al. [58]. CT images (Fig 6) reveal no differences between the bone surrounding the oval cavity and the other bone of the dentary. So, no evidence of thickened bone, bone expansions, bone fusions, high density bone tissues, bone eroded, resorption of bone tissue or other abnormal bone formations, can be observed on CT images.

Analysing the pathologies present in the literature affecting dinosaurs (S1 File), we could see if any of these pathologies can be assigned as responsible for the formation of the aforementioned oval hole in our dentary. In the case of MQ98-II-1 specimen, we can exclude those diseases that involve growth or regrowth of the bone since what we find is a deep oval cavity, not an expansion of the bone. So, we can rule out as our “smoking gun” these diseases: bony callus, bony growths, bone hyperostosis, chondrosarcoma, diffuse hydropathic skeletal hyperostosis, exostosis, ossifying fibroma, osteochondroma, osteoma, osteomyelitis, osteosarcoma and Paget’s disease. We can also discard those pathologies that involve deformation of the bone, its fusion or fractures, such as: ankylosing spondylitis, bone deformity, bones fused together, pseudarthrosis or stress fracture.

Therefore, only a few diseases can produce pathologies compatible with the presence of an oval cavity similar to the one found in MQ98-II-1. These are: ameloblastoma, bone resorption, gout, haemangioma, Langerhans cell histiocytosis, multiple myeloma and osteoblastoma.

Among these pathologies, oval cavity in Portell dentary can be distinguished from: ‘honeycomb’ or ‘soap bubble’ appearance produced by internal septations in ameloblastoma. It tends to be expansive, producing exostosis usually separated from the normal trabecular bone by a thin layer of longitudinally expanded bony [62]. Also differs from pitting areas in bones due to mineral releasing produced by resorption of bone tissue that can produce osteoporosis. No correlation with sclerotic-rimmed lesions produced by gout are observed. No trace of the characteristic linear residual trabeculae and the bubbly aspect produced by haemangiomas. Langerhans cell histiocytosis produces a multiple lesions coalescence (“geographic” appearance), “space-occupying masses” and effaced trabeculae, not present in MQ98-II-1. In multiple myeloma, local disappearance of normal bone due to resorption provokes an expansile spherical appearance, penetrating cortical as well as trabecular bone. Finally, no signs of sclerotic margins and very fine trabeculae like in osteoblastomas, are present in the dentary.

A large amount of paleopathologies present in dinosaur specimens have been revised in order to find any similar structure such as this oval cavity present in MQ98-II-1 (21 mm long and 9 mm wide). We only found evidences of a 20 mm round hole in the jugal and epijugal of *Pachyrhinosaurus* (TMP 86.55.304 specimen), but Tanke and Rothschild [63] considered this hole as formed as a result of non-pathological processes.

On the other hand, in extinct dinosaurs (non-avian) the most common diseases are fractures and traumas and we can exclude them as the cause of the hole in Portell dentary. Infections (post-traumatic and non-traumatic) are the second cause of diseases in dinosaurs, but none of them are responsible of a similar pathology. Neoplastic disease and metabolic disorders are rare in vertebrate fossils [58, 59, 61], so it is highly unlikely that they are the cause of such structure in the dentary.

Following Hamm et al. [58], we search for pathologies found in close relatives, that help us to increase the level of confidence for this diagnosis, and we could not find evidences of infections or neoplastic disease in Iguanodontia (except in Hadrosauridae). In contrast, we could find pathologies as a result of fractures and/or traumas (e.g.: osteoarthritis [64] or calcifications [65] in *I*. *bernissartensis*).

Furthermore, the cavity is the continuation of a channel (coronoid process medial groove) that separates the inner edge of the coronoid process from the outer ones, so it seems an anatomical character.

Clearly, we can conclude: it is more plausible that oval cavity present in MQ98-II-1 is an apomorphic character rather than a paleopathology.

## Phylogenetic analysis

### Methodology

A phylogenetic analysis was conducted using a modified version of Verdú et al. [7] data set (S2 and S3 Files) based on 148 character list (S4 File). We revised the data set in its matrix and we added some missing jaw characters to the specimens of *Barilium dawsoni* [47], *Bolong yixianensis* [39], and *Hypselospinus fittoni* [48]. In addition to, we revised and modified some others to *Hypselospinus fittoni* [48], *Lanzhousaurus magnidens* [33], *Mantellisaurus* [38], *Ouranosaurus* [31], *Owenodon* [37], and *Proa* [11] (S5 File). Also, we added to the data set the characters of *Magnamanus soriaensis* [49].

The matrix includes 66×148 (66 operational taxonomic units [OTUs] and 148 cranial and postcranial characters). For *Portellsaurus sosbaynati* gen. et sp. nov. 13 (cranial) characters were scored from the available information from the specimen (holotype dentary MQ98-II-1). This modified matrix was analysed using TNT v.1.5 software [66].

*Lesothosaurus diagnosticus*, as an ornithischian outgroup, and *Hypsilophodon foxii*, as a non-iguanodontian ornithopod, were employed as constraints. We conducted two runs, one with all characters unordered (as in Verdú et al. [7], McDonald [2], and McDonald et al. [67]) and second ones with twelve characters ordered (as in McDonald [2]).

### Characters unordered

**Fig 7:**
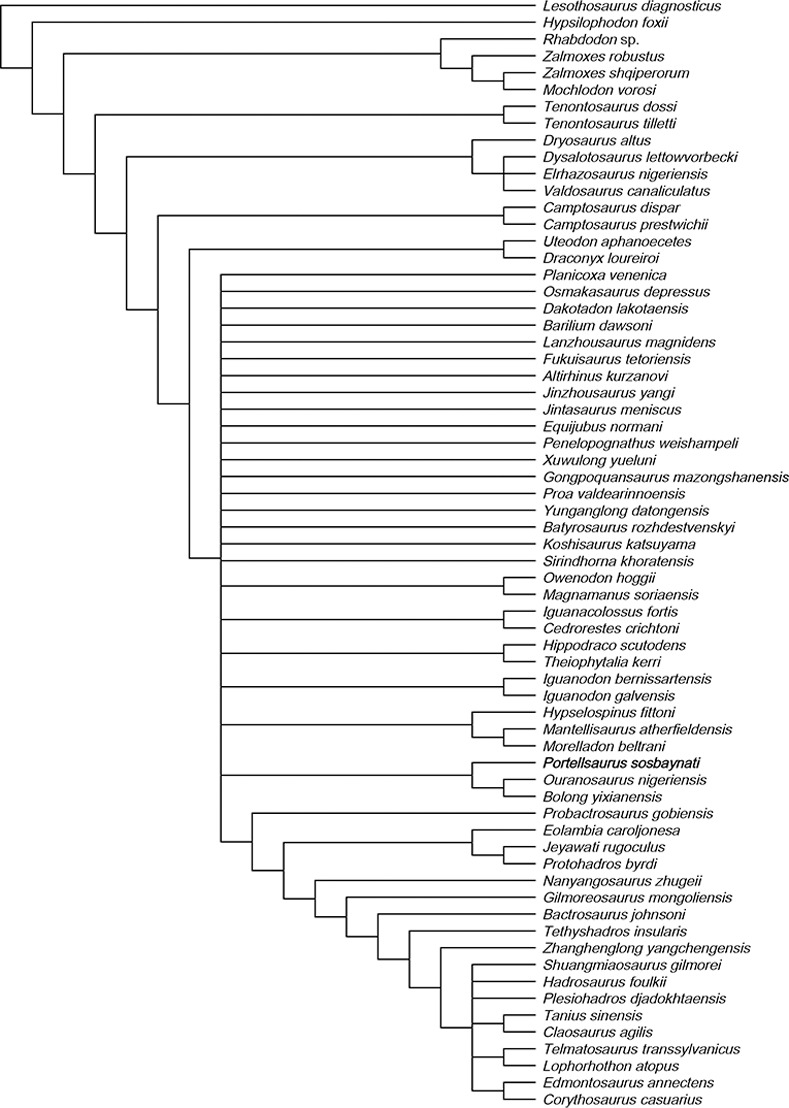
Phylogenetic relationships of *Portellsaurus sosbaynati* gen. et sp. nov. 50% majority rule consensus of 440 most parsimonious trees.

In this case, we perform a “traditional search” with the tree bisection reconnection algorithm in TNT [66]. Wagner trees with a random seed of 1; 9,999 replicates with 10 trees saved per replication were used. All characters were equally-weighted and treated as unordered. TNT examined 4,782,642,737 rearrangements. As a result, 440 MPTs (Most Parsimonious Trees) were recovered. The strict consensus tree calculated with TNT is poorly resolved with a length of 936 steps, a consistency index (CI) of 0.2318, a homoplasy index (HI) of 0.7682, and a retention index (RI) of 0.4077 (the ‘describetree’ command in PAUP 4.0a build 167 [68]). After the application of a 50%-majority rule, the resultant tree is more resolved (Fig 7) and it has a length of 651 steps (CI = 0.3333, HI = 0.6667, RI = 0.6425).

### Characters ordered

**Fig 8:**
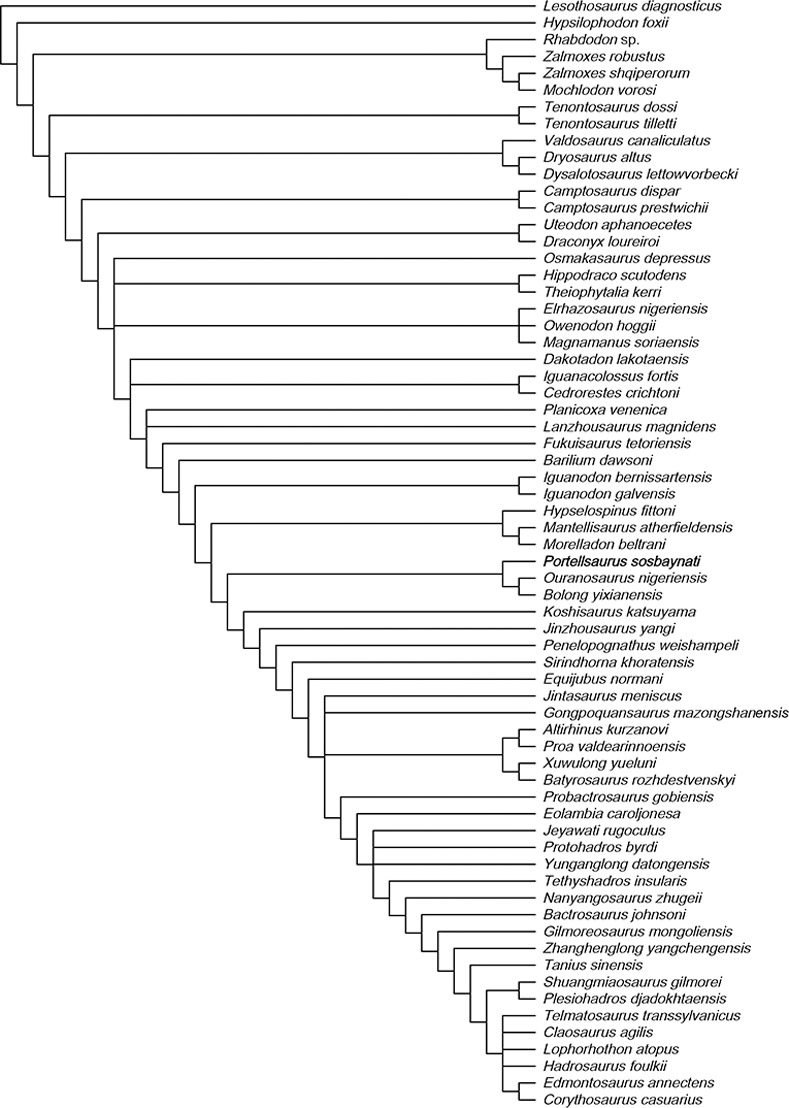
Phylogenetic relationships of *Portellsaurus sosbaynati* gen. et sp. nov. 50% majority rule consensus of 30 most parsimonious trees.

A second “traditional search” with the same search parameters used in the first analysis were performed. Twelve multistate characters (10, 14, 20, 25, 46, 67, 81, 82, 83, 100, 127, and 130) were treated as ordered (additive in TNT, as in McDonald [2]) using the method of intermediates proposed by Wilkinson [69]. TNT examined 4,448,921,232 rearrangements, and it finds 30 MPTs (Most Parsimonious Trees). As in the previous analysis, the strict consensus tree is poorly resolved with a length of 705 steps (CI = 0.3078, HI = 0.6922, RI = 0.6176). After the application of a 50%-majority rule, the resultant tree is more resolved (Fig 8) and it has a length of 535 steps (CI = 0.4056, HI = 0.5944, RI = 0.7508).

### Phylogenetic results

As Pol and Escapa [70] and Pol and Goloboff [71] notate, paleontological datasets often have lot of missing data, that leading to obtaining multiple MPTs and a collapsed strict consensus. It is reflected in strict consensus trees with the presence of polytomies. Several methods have been used for identifying these unstable taxa in optimal trees [70, 71] and we use the Pol and Escapa methodology [70] to find these rogue taxa both in ours ordered an unordered characters data set. This detects unstable branches and scores particular characters related to their instability. The analysis performs an iterative examination of the agreement of triplets among the optimal topologies (MPTs) and it evaluates the character optimizations on these trees. The process could be done through a TNT script (IterPCR.run) [70]. It returns a list of characters that they show the instability of every unstable taxon. All the unstable taxa are identified and pruned from the tree.

**Fig 9:**
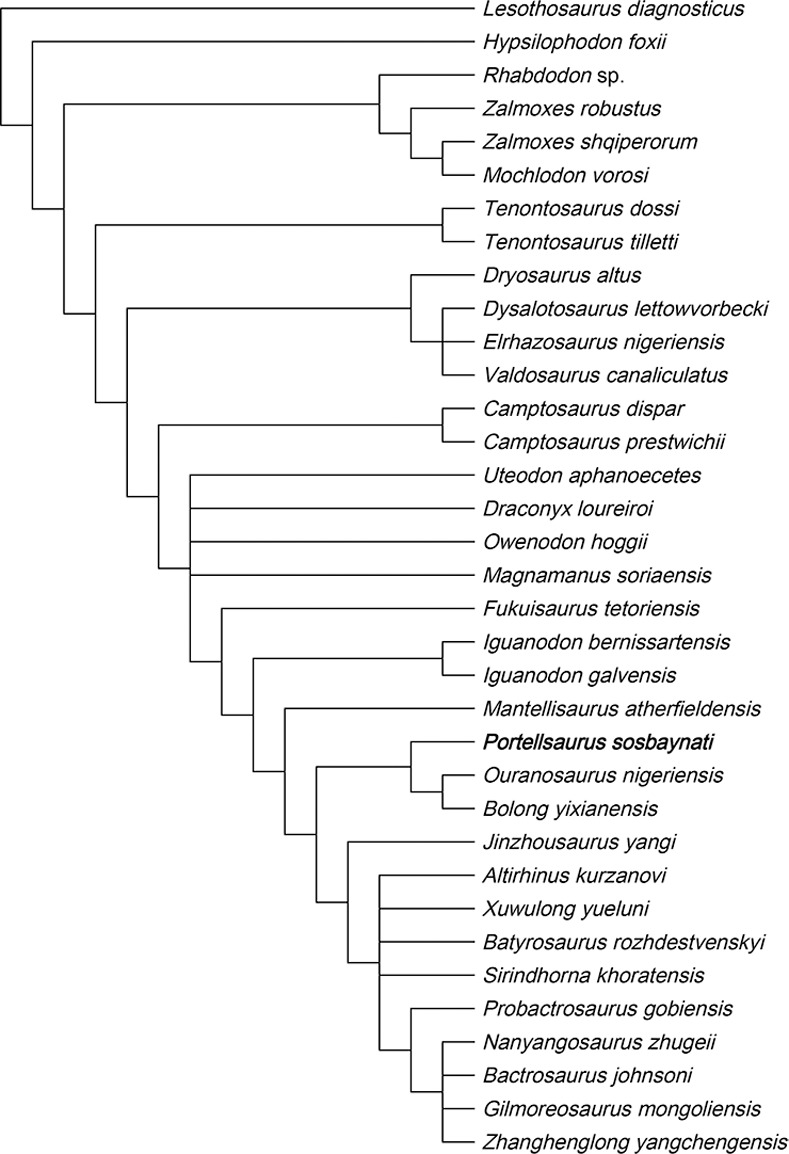
Phylogenetic relationships of *Portellsaurus sosbaynati* gen. et sp. nov. Strict reduced consensus obtained after running the IterPCR script in the MPTs from unordered dataset.

After the iterative PCR procedure on the unordered dataset, we obtain a most-parsimonious tree with a length of 382 steps (CI = 0.5340, HI = 0.4660, RI = 0.7201). The strict consensus tree produces one major polytomy (degree 50) and the Iterative PCR identified 38 unstable branches (37 taxa 1 clade) after 12 iterations, 30 of which were pruned (Fig 9).

**Fig 10:**
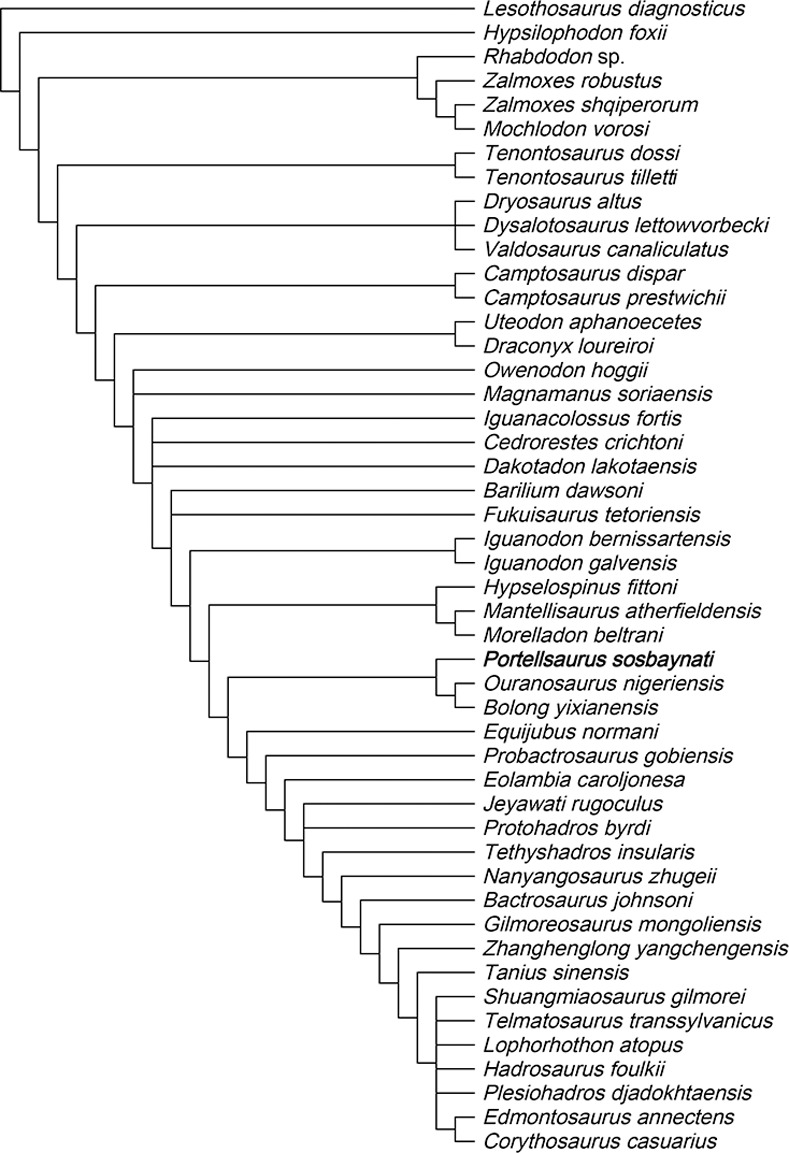
Phylogenetic relationships of *Portellsaurus sosbaynati* gen. et sp. nov. Strict reduced consensus obtained after running the IterPCR script in the MPTs from ordered dataset.

On the other hand, after the same procedure on the ordered dataset, we obtain a most-parsimonious tree with a length of 461 steps (CI = 0.4664, HI = 0.5336, RI = 0.7745). The strict consensus tree produces 4 major polytomies (1 polytomy degree 6, 1 polytomy degree 8, 1 polytomy degree 15 and 1 polytomy degree 19) and the Iterative PCR identified 25 unstable branches (24 taxa 1 clade) after 6 iterations, 17 of which were pruned (Fig 10).

From the six calculated consensus trees, *Portellsaurus* is consistently well resolved among five of them, where it always appears next to *Ouranosaurus* and *Bolong*. It is important to note that after applying the IterPCR script none of these taxa have been eliminated from the strict reduced consensus trees (Figs 9 and 10). The aim of this methodology is to objectively evaluate the unstable taxa present in polytomies of the strict consensus trees. This instability is caused either by the lack of information or by incongruent scoring of characters. In the case of *Portellsaurus* the results of the IterPCR shows the stability of the taxon in the strict consensus.

Finally, to analyse the phylogenetic relationships of *Portellsaurus sosbaynati* gen. et sp. nov. with other taxa, we have taken the strict reduced consensus tree obtained after running the IterPCR script from the ordered matrix, since it still conserves 48 of the 66 original taxa and it presents a well-resolved consensus.

The ‘Bremer Support’ analysis was conducted using TNT software and the bootstrap resampling was performed using PAUP software following the methodology proposed by Prieto-Márquez [72] for the hadrosaurid phylogeny: 5000 replicates using a ‘heuristic search’, each search being conducted using ‘random additional sequences’ with ‘branch-swapping by Subtree Pruning and Regrafting’ (SPR) and 25 replicates. The maximum number of trees recovered per replicate was 100 (the default memory setting). We used TNT software to identify which synapomorphies supported each node. Only unambiguous synapomorphies were calculated, and branches with no possible support were collapsed ("rule 3"). This makes results more conservative.

**Fig 11:**
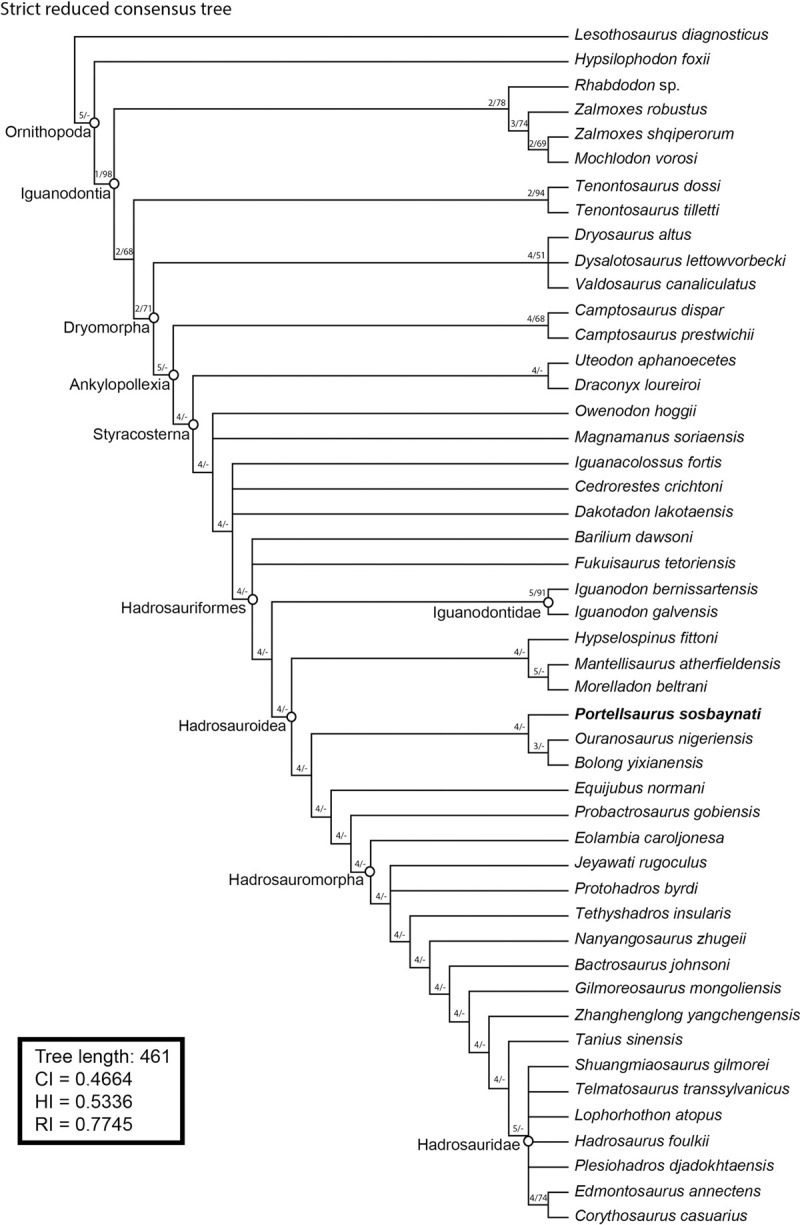
Phylogenetic relationships of *Portellsaurus sosbaynati* (MQ98-II-1). Strict reduced consensus (IterPCR script) from ordered dataset of 66 OTUs with 461 steps (CI = 0.4664, HI = 0.5336, RI = 0.7745). Notes: Numbers at the left of the node indicate, first, the decay index (Bremer support) and, second, the bootstrapping score obtained for such a node. A hyphen (-) indicates that the node has not been recovered in either the Bremer support analysis or bootstrapping. The phylogenetic position of *Portellsaurus sosbaynati* is indicated in bold.

In this tree (Fig 11), *Barilium dawsoni* and *Fukuisaurus tetoriensis* are the most basal members of Hadrosauriformes in the current analysis. We consider the superfamily Iguanodontoidea (*sensu* Verdú et al. [7]) an inactive taxon following Sereno [24]. Our phylogenetic analysis is inconsistent with the definition of this superfamily. Alternatively, our results support the presence of the family Iguanodontidae (Fig 11), defined as the most inclusive monophyletic clade containing *Iguanodon bernissartensis* but not *Corythosaurus casuarius*. In the present hypothesis, Iguanodontidae is formed exclusively by European Barremian–Aptian taxa and has the topology (*I*. *bernissartensis*, *I*. *galvensis*). The apomorphic characters that support Iguanodontidae are listed below:

94—Caudal margin of scapula straight, dorsal and ventral margins are parallel approaching the caudal margin of the scapula and meet the caudal margin at nearly right angles (unambiguous).
114—Femur, the distal half of the shaft curved caudally in lateral view (unambiguous).
132—The midpoint of the quadratojugal notch is located ventral to the midpoint of the quadrate height (unambiguous).
135—Seven or more sacral vertebrae (unambiguous).
137—Absence of the coracoid foramen on its external surface, a notch opens between the glenoid and the scapular process (unambiguous).

**Fig 12:**
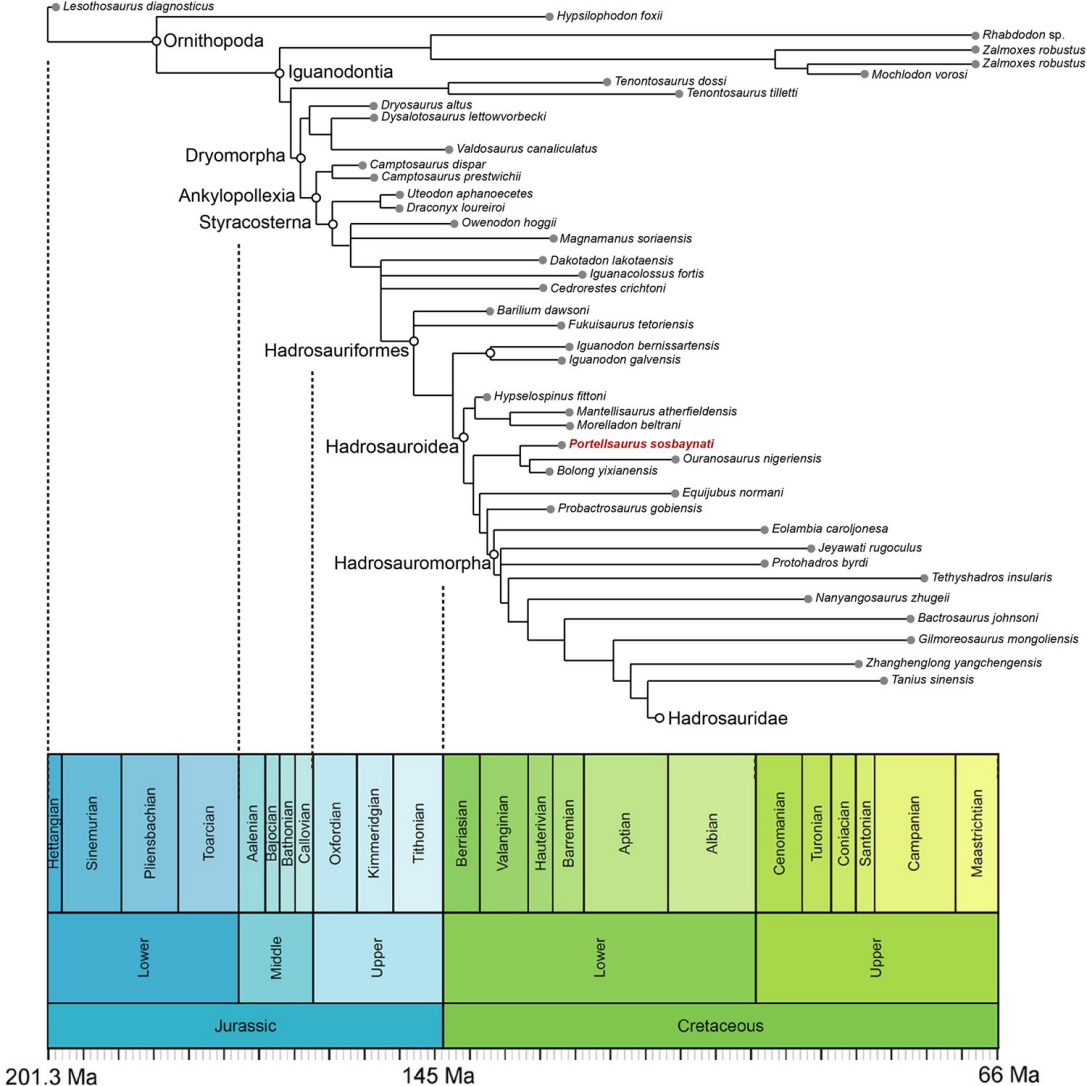
Time-calibrated relationships of *Portellsaurus sosbaynati* (MQ98-II-1). Time-calibrated strict reduced consensus tree resulting from the analysis of the modified data matrix from Verdú et al. [7]. Each taxon is plotted stratigraphically based on its first appearance date.

*Portellsaurus sosbaynati* is recovered as the sister taxon of *Ouranosaurus* + *Bolong* (Figs 11 and 12). Unambiguous synapomorphies among *Portellsaurus*, *Ouranosaurus*, and *Bolong* include characters 14 and 20, which correspond to a: (14) caudal-most extent of tooth row situated medial to the coronoid process but still rostral to the longitudinal axis of the process; (20) caudally-oriented inclined coronoid process.

The apomorphic character that supports *Portellsaurus sosbaynati* gen. et sp. nov. is (17) absence of bulge along the ventral margin directly ventral to the base of the coronoid process (unambiguous).

## Conclusions

This work describes and discusses the type specimen (MQ98-II-1) of the styracosternan hadrosauroid *Portellsaurus sosbaynati* gen. et sp. nov. from the Mirambell Formation, early Barremian (Early Cretaceous) Mirambell formation of Portell (Castellón, Spain). The holotype is described based upon cranial element, a nearly complete right dentary, of a large individual (6 to 8 m).

Two autapomorphies support the validity of the type species. In addition, *Portellsaurus sosbaynati* gen. et sp. nov. can be distinguished from other styracosternan hadrosauroids on the basis of a unique combination of characters. Regardless of the phylogenetic analysis carried out, it is clearly nested within the clade that contains *Ouranosaurus* and *Bolong*.

Taking into account the works of Norman [45], McDonald [2] and Verdú et al. [7], we can state that *Portellsaurus sosbaynati* has more similarities with *Ouranosaurus nigeriensis* and *Bolong yixianensis* than with other hadrosauriforms like *I*. *bernissartensis*, *I*. *galvensis*, *Proa valdearinnoensis*, *Koshisaurus katsuyama*, *Hypselospinus fittoni*, and *Mantellisaurus atherfieldensis*. The morphology of the dentary from Portell is sufficiently different from these last-mentioned taxa, as it has shown in this work, to be considered a new taxon. Despite the similarities it offers with *Ouranosaurus* and *Bolong*, it is highly unlikely that the specimen from the early Barremian of Spain, was the same taxon as the late Barremian–early Aptian of China or the late Aptian of Niger.

Although dinosaur sites from the Margas de Mirambell Formation (early Barremian–early late Barremian) are abundant [73, 74], to date it has not been possible to determine the presence of dinosaurs beyond the identification at the family and/or subfamily level [73, 74]. This is because, scarce material has been found in this geological formation. So, studies and publications are few. This is in contrast to the fact that the presence of other hadrosauriform dinosaurs have been described at a specific level in the Morella sub-basin e.g. *Iguanodon bernissartensis*, *Mantellisaurus atherfieldensis*, and *Morelladon beltrani* from the Arcillas de Morella Formation (late Barremian) of Morella (Castellón), or in neighbouring sub-basins, e.g. the Galve sub-basin: *Iguanodon galvensis* from the Camarillas Formation (early Barremian) of Galve (Teruel) and the Oliete sub-basin: *Proa valdearinnoensis* from the Escucha Formation (early Albian) of Ariño (Teruel). Hence, *Portellsaurus sosbaynati* gen. et sp. nov. is definitely the first styracosternan dinosaur species identified from the Margas de Mirambell Formation (early Barremian–early late Barremian) in the Morella sub-basin (Maestrat Basin, eastern Spain).

## Supporting information

S1 File Paleopathologies. (DOCX)

S2 File Data matrix. (TXT)

S3 File Data matrix. (NEX)

S4 File Character list. (DOCX)

S5 File Data set modifications. (DOCX)
