# Supplementary material for: A new styracosternan hadrosauroid (Dinosauria: Ornithischia) from the Early Cretaceous of Portell, Spain
Source: PLoS One. 2021 Jul 7;16(7):e0253599. doi: 10.1371/journal.pone.0253599 (PMC8262792; doi:10.1371/journal.pone.0253599)
Supplement: S1 File — (DOCX) [file pone.0253599.s001.docx]

**Supporting information 1 – Paleopathologies**

Below we present a table with different pathologies present in dinosaur and their characteristics.

References can be found at the end of this document.

**Summary table**

| **Paleopathology** | **Characteristics** |
| --- | --- |
| Ameloblastoma | Benign tumor with a strong preponderance for the mandible. Internal septations produce a ‘honeycomb’ or ‘soap bubble’ appearance. |
| Ankylosing spondylitis | Two or more vertebrae growing closer together and even fuse. New rigid bone tissue that affected joints is deposited. |
| Bony callus | It is comprised of two adjoining but distinct types of bone. There is woven bone and lamellar bone. |
| Bone deformity | A bone that is not the normal shape or size. It may also be positioned incorrectly which causes poor alignment. |
| Bones fused together | Two or more bones fused together. |
| Bony growths | These lesions give the bone a very rough texture and deform. |
| Bone hyperostosis | An excessive growth of bone, that may lead to exostosis. |
| Bone resorption | Resorption of bone tissue, which osteoclasts break down the tissue in bones and release the minerals. It increases pit numbers and pit areas in the bone, and it can produce osteoporosis. |
| Chondrosarcoma | Uncontrolled growth of cancerous cells that produce cartilage. |
| Diffuse hydropathic skeletal hyperostosis | Characteristic ossification patterns that can occur in the spine and peripheral entheses. |
| Exostosis (bone spur) | Formation of new bone on the surface of a bone. |
| Gout | Metabolic disorder in which urate crystals accumulate as space-occupying masses, producing monarticular spheroidal erosions in bone. |
| Haemangioma | Vascular tumors which may be bubbly or have characteristic linear residual trabeculae. |
| Langerhans Cell Histiocytosis | Multiple lesions coalescence (“geographic” appearance), “space-occupying masses” and effaced trabeculae. |
| Multiple myeloma | Appears as "lytic lesions" with local disappearance of normal bone due to resorption. |
| Ossifying fibroma | Prominent calcified structures (ossicles and cementicles) that appeared as eosinophilic or basophilic spherules of osteoid or bone within a moderately cellular, dense stroma. |
| Osteoblastoma | Large expansile lytic lesions, very circumscribed lesions with sclerotic margins and very fine trabeculae. |
| Osteochondroma | Cartilage-capped bony projections or outgrowth on the surface of bones exostoses. |
| Osteoma | New piece of bone growing on another piece of bone. |
| Osteomyelitis | Infective process that encompasses all of the bone components. It can lead to bone sclerosis and deformity. New bone is deposited around the area of necrosis. |
| Osteosarcoma | Characterised by the direct formation of immature bone or osteoid tissue by the tumour cells. Often presents a Codman's triangle. |
| Paget’s disease | Enlargement and deformity of bones due to a combination of abnormal bone resorption and abundant new bone formation. |
| Pseudoarthrosis (false joint) | A fracture with nonunion. |
| Stress fracture | A fatigue-induced bone fracture caused by repeated stress over time. It can be described as small cracks in the bone. |

**References**

Dumbravă MD, Rothschild BM, Weishampel DB, Csiki-Sava Z, Andrei RA, Acheson KA, Codrea VA. A dinosaurian facial deformity and the first occurrence of ameloblastoma in the fossil record. Scientific Reports. 2016; 6(29271). https://doi.org/10.1038/srep29271

Hamm CA, Hampe O, Schwarz D, Witzmann F, Makovicky PJ, Brochu CA, Reiter R, Asbach P. A comprehensive diagnostic approach combining phylogenetic disease bracketing and CT imaging reveals osteomyelitis in a Tyrannosaurus rex. Scientific Reports. 2020; 10(18897). https://doi.org/10.1038/s41598-020-75731-0

McWhinney L, Carpenter K, Rothschild B. Dinosaurian humeral periostitis: a case of a juxtacortical lesion in the fossil record: In: Tanke DH, Carpenter K, editors. Mesozoic Vertebrate Life. Bloomington: Indiana University Press; 2001. pp. 364–377.

Molnar RE. Theropod paleopathology: a literature survey. In: Tanke DH, Carpenter K, editors. Mesozoic Vertebrate Life. Bloomington: Indiana University Press; 2001. pp 337–363.

Rega E, Holmes R, Tirabasso A. Chapter 24: Habitual Locomotor Behavior Inferred from Manual Pathology in Two Late Cretaceous Chasmosaurine Ceratopsid Dinosaurs, Chasmosaurus irvinensis (CMN 41357) and Chasmosaurus belli (ROM 843). In: Ryan MJ, Chinnery-Allgeier BJ, Eberth DA, editors. New Perspectives on Horned Dinosaurs: The Royal Tyrrell Museum of Paleontology Ceratopsian Symposium. Bloomington: Indiana University Press; 2010. pp. 340–354.

Rothschild BM. Radiologic assessment of osteoarthritis in dinosaurs. Annals of Carnegie Museum. 1990; 59:295–301.

Rothschild BM, Tanke DH, Helbling M, Martin LD. Epidemiologic study of tumors in dinosaurs. Naturwissenschaften. 2003; 90:495–500.

Rothschild BM, Tanke DH, Rühli F, Pokhojaev A, May H. Suggested Case of Langerhans Cell Histiocytosis in a Cretaceous dinosaur. Scientific Reports. 2020; 10(2203). https://doi.org/10.1038/s41598-020-59192-z

Tanke DH, Rothschild BM. DINOSORES: An Annotated Bibliography of Dinosaur Paleopathology and Related Topics. New Mexico Museum of Natural History and Science. 2002; 20:1838–2001.

Tanke, DH, Rothschild BM. Chapter 25: Paleopathologies in Albertan Ceratopsids and Their Behavioral Significance. In: Ryan MJ, Chinnery-Allgeier BJ, Eberth DA, editors. New Perspectives on Horned Dinosaurs: The Royal Tyrrell Museum of Paleontology Ceratopsian Symposium. Bloomington: Indiana University Press; 2010. pp. 355–384.
