# Supplementary material for: A new styracosternan hadrosauroid (Dinosauria: Ornithischia) from the Early Cretaceous of Portell, Spain
Source: PLoS One. 2021 Jul 7;16(7):e0253599. doi: 10.1371/journal.pone.0253599 (PMC8262792; doi:10.1371/journal.pone.0253599)
Supplement: S4 File — (DOCX) [file pone.0253599.s004.docx]

**Supporting information 2 - Character list**

We realized a phylogenetic analysis using original characters from Verdú et al. (2018). Below we present the list of characters used in the matrix. Matrix is in the Supporting information 1 (S1_File). References can be found at the end of this document.

**0 - Predentary**, overall shape of oral portion in dorsal view (modified from Weishampel *et al*. 2003, character 18; and Prieto-Márquez et al. 2006, character 5):

1. Subtriangular, comes to a point without a distinct rostral portion.
2. Arcuate, rounded rostrolateral corners.
3. Subrectangular, squared corners and straight, well demarcated rostral portion.

**1 - Predentary**, directions of lateral margins of lateral processes relative to each other in dorsal and ventral views:

- 1. Divergent.
  2. Parallel.

**2 - Predentary**, morphology of ventromedial process (Weishampel et al. 2003, character 20):

1. Undivided.
2. Bifurcated.

**3 - Predentary**, dorsomedial process:

- 1. Absent.
  2. Present.

**4 - Predentary**, denticles (Weishampel *et al*. 2003, character 19):

1. Absent.
2. Present.

**5 - Predentary,** denticle morphology (modified from Prieto-Márquez 2010, characters 25 and 27):

1. Large, conical median denticle with one or two prominent conical denticles of subequal size adjacent to the median denticle on both sides and smaller, tab-like denticles on lateral processes.
2. Rostrocaudally compressed prong-like denticles that increase in size towards the midline of the predentary.
3. Rostrocaudally compressed prong-like denticles of equal size.

**6 - Predentary**, grooves on either side of midline on rostral surface, extending ventrolaterally to dorsomedially:

1. Absent.
2. Present.

**7 - Dentary**, orientation of symphysis relative to lateral margin of dentary (Prieto-Márquez et al. 2006, character 10):

1. Rostrolateral to caudomedial (medial edge of symphysis and lateral margin of dentary diverge in dorsal view).
2. Parallel.

**8 - Dentary**, orientation of symphyseal region in lateral view:

1. Ventral surface of symphyseal region visible in lateral view, articulation site for ventromedial process of predentary faces ventolaterally.
2. Ventral surface of symphyseal region not visible in lateral view, articulation site for ventromedial process of predentary faces ventrally.

**9 - Dentary**, diastema:

1. Absent.
2. Present.

**10 - Dentary**, shape of tooth row in dorsal view (modified from Prieto-Márquez et al. 2006, character 8):

1. Bowed medially at mid-length.
2. Bowed medially along caudal half.
3. Straight.

**11 - Dentary**, shape of tooth row in lateral view:

1. Straight.
2. Concave.
3. Convex.

**12 - Dentary**, orientation of tooth row relative to lateral surface of dentary:

1. Convergent rostrally and caudally.
2. Convergent rostrally and divergent caudally.

**13 - Dentary**, morphology of tooth alveoli (Norman 2002, character 33):

1. Alveoli shaped by dentary teeth.
2. Alveoli with parallel vertical walls.

**14 - Dentary**, caudal-most extent of tooth row (modified from You et al. 2003, character 29):

1. Rostral to base of coronoid process.
2. Medial to coronoid process but still rostral to longitudinal axis of the process.
3. Even with longitudinal axis of the coronoid process.
4. Caudal to longitudinal axis of the coronoid process but still rostral to the caudal margin of the process.
5. Caudal to the base of the coronoid process.

**15 - Dentary,** shape in lateral or medial view (modified from Norman 2004, character 22):

1. Dorsal and ventral margins converge.
2. Dorsal and ventral margins are parallel.
3. Dorsal and ventral margins diverge, the dentary deepens rostrally.

**16 - Dentary**, morphology of ventral margin of rostral ramus leading to the predentary articulation:

1. Straight.
2. Ventral margin inflected ventrally, such that it curves gently towards the predentary articulation and symphysis.
3. Ventral margin curves dorsally towards symphysis.

**17 - Dentary**, bulge along ventral margin directly ventral to the base of the coronoid process (modified from Prieto-Márquez 2010, character 41):

1. Absent.
2. Present.

**18 - Dentary**, bulge on the lateral surface ventral to the coronoid process that gives rise to the process (modified from Prieto-Márquez 2010, character 46):

1. absent.
2. present.

**19 - Dentary**, platform between the tooth row and the coronoid process (modified from Norman 2002, character 26):

1. Absent, tooth row curves into base of coronoid process.
2. Present.

**20 - Dentary**, orientation of coronoid process (modified from Prieto-Márquez et al. 2006, character 7):

1. Caudally inclined.
2. Vertical.
3. Rostrally inclined.

**21 - Dentary**, expansion of dorsal end of coronoid process:

1. Absent.
2. Present.

**22 - Dentary**, expansion of dorsal end of coronoid process, location (modified from McDonald et al. 2010, character 33):

1. Along rostral edge only.
2. Along rostral and caudal edges.

**23 - Dentary**, position of greatest rostrocaudal width of expanded coronoid process:

1. ventral to apex.
2. at apex.

**24 - Surangular**, surangular foramen (modified from Weishampel et al. 1993, character 27):

1. Present.
2. Absent.

**25 - Surangular**, external mandibular fenestra (modified from Kobayashi & Azuma 2003, character 15):

1. Large, open fenestra between dentary, surangular, and angular.
2. Small foramen (“accessory foramen”) on surangular near suture with dentary.
3. Absent.

**26 - Surangular**, shape of contact with angular in lateral view:

1. Inclined rostrodorsal to caudoventral.
2. Sinuous.
3. Horizontal.

**27 - Angular**, exposure in lateral view (modified from Norman 2002, character 28):

1. Present, groove on ventral margin of surangular for articulation with angular.
2. Absent, articulation with surangular occurs on the medial surface of that bone.

**28 - Premaxilla**, morphology of rostral margin in dorsal view:

1. Premaxillae not transversely expanded, snout comes to a point.
2. Premaxillae laterally expanded, snout squared.

**29 - Premaxilla**, tooth alveoli:

1. Present.
2. Absent.

**30 - Premaxilla**, ventral inflection (modified from Norman 2002, character 2):

1. Absent, oral margin even with ventral margin of maxilla.
2. Present, oral margin projects farther ventrally than ventral margin of maxilla.

**31 - Premaxilla**, morphology of caudolateral corner of oral margin in lateral view:

1. In contact with maxilla.
2. Free and gently curved.
3. Free and angular.

**32 - Premaxilla**, everted rim on lateral edge of oral margin (modified from Weishampel et al. 1993, character 3):

1. Absent.
2. Present.

**33 - Premaxilla**, denticles on oral margin (modified from Weishampel et al. 2003, character 7):

1. Absent.
2. Present.

**34 - Premaxilla**, denticle morphology:

1. One large conical denticle adjacent to interpremaxillary suture on each premaxilla.
2. Two large, rostrocaudally elongate denticles on each premaxilla.
3. Four or more conical denticles of similar size on each premaxilla.
4. Three rostrally projecting denticles that decrease in size laterally.

**35 - Premaxilla**, morphology of caudal ramus of ventrolateral process (modified from Prieto-Márquez 2010, character 71):

1. Tapers to a point.
2. Dorsoventrally expanded.

**36 - Premaxilla**, contact with lacrimal (Weishampel et al. 2003, character 8):

1. Absent.
2. Present.

**37 - Premaxilla**, contact with prefrontal:

1. Absent.
2. Present.

**38 - External naris**, position (modified from Weishampel et al. 2003, character 2):

1. Confined to area immediately above oral margin of premaxilla.
2. Extends caudally so as to lie dorsal to maxilla.

**39 - Maxilla**, bifurcated rostral end (modified from Prieto-Márquez et al. 2006, character 17):

1. Absent.
2. Present.

**40 - Maxilla**, direction of rostroventral process:

1. Rostrally directed.
2. Rostroventrally curved.

**41 - Maxilla**, ventral margin of tooth row in lateral view:

1. Straight.
2. Concave.

**42 - Maxilla**, shape in dorsal view:

1. Bowed medially.
2. Straight for most of length.
3. Bowed laterally.

**43 - Maxilla**, shape of tooth row in ventral view:

1. Medially bowed, with rostral and caudal ends curving laterally.
2. Bowed laterally.
3. Straight.

**44 - Maxilla**, shape of ascending process:

1. Rostrocaudally narrow and hook-like.
2. Rostrocaudally broad and subtriangular.

**45 - Maxilla**, jugal process morphology (modified from Norman 2002, character 15):

1. Dorsally concave, rostrodorsally to caudoventrally inclined shelf, scarf contact with jugal.
2. Sinuous shelf, scarf contact with jugal.
3. Caudolaterally projecting jugal process, “finger-in-recess” contact with jugal.
4. Mediolaterally broad, flat surface against which jugal abuts.

**46 - Maxilla**, antorbital fossa, extent in lateral view:

1. Occupies most of lateral surface of ascending process.
2. Rostrocaudally elongate, elliptical depression restricted to caudal half of ascending process.
3. Small semicircular depression restricted to caudal margin of ascending process.
4. Antorbital fossa not visible in lateral view.

**47 - Lacrimal**, contact with nasal (Norman 2002, character 12):

1. Present.
2. Absent.

**48 - Postorbital**, shape of caudal end of squamosal process that overlaps the lateral surface of the squamosal modified from Prieto-Márquez 2010, character 132):

1. Tapers to a point.
2. Irregularly rounded.
3. Bifurcated.

**49 - Jugal**, articulation with ectopterygoid (Head 1998, character 6):

1. Present.
2. Absent.

**50 - Jugal,** morphology of portion of maxillary process that overlaps maxilla (modified from Norman 2002, character 14):

1. Tapers at rostral ends of maxillary and lacrimal contacts, practically straight, with slightly convex ventral margin and slightly concave dorsal margin.
2. Subrectangular elongated process with nearly parallel dorsal and ventral margins.
3. Tapers with sinuous dorsal and ventral margins.
4. Dorsoventrally expanded toward the maxilla contact.
5. Dorsoventrally expanded to form part of rostral margin of orbit, but abruptly truncated.

**51 - Jugal**, shape of free ventral margin caudal to maxillary and ectopterygoid contacts (modified from Norman 2002, character 16):

1. Straight.
2. Sinuous, jugal dorsoventrally expanded ventral to infratemporal fenestra.
3. Sinuous with striated, caudally-directed flange that projects caudal to jugal-quadratojugal contact.
4. Angular, with prominent ventrally-directed flange ventral to infratemporal fenestra.
5. Dorsoventrally narrow and strap-like, with convex ventral margin and concave dorsal margin that are parallel.

**52 - Jugal**, contribution of caudal ramus to ventral margin of infraorbital fenestra (Weishampel et al. 2003, character 11):

1. Partial, quadratojugal also forms part of margin.
2. Caudal ramus forms entire ventral margin of infraorbital fenestra.

**53 - Quadratojugal**, quadratojugal foramen (Weishampel et al. 2003, character 17):

1. Absent.
2. Present.

**54 - Quadrate**, quadratojugal notch in lateral wing (McDonald et al. 2010, character 16):

1. Absent.
2. Present.

**55 - Quadrate**, morphology of the lateral profile of the quadratojugal notch of the quadrate (from Prieto-Márquez 2010, character 119):

1. Subcircular, with a ventral half of the notch that is recurved and has a horizontal rostral segment.
2. Wide arcuate and asymmetrical, with the ventral half of the notch having a short horizontal rostral segment.
3. Wide arcuate and symmetrical, with the ventral half of the notch being rostroventrally-directed and nearly straight, as it is in the dorsal half.

**56 - Quadrate**, paraquadrate foramen:

1. Absent, caudal margin of quadratojugal contacts entire rostral margin of quadrate along the contact surface.
2. Present, gap between portion of caudal margin of quadratojugal and rostral margin of quadrate.

**57 - Quadrate**, overall shape in lateral or medial view:

1. Curved gently caudally along entire length.
2. Straight for much of dorsoventral length, curved caudally near dorsal end.
3. Straight.

**58 - Quadrate**, shape of dorsal condyle:

1. Subtriangular, broad rostral margin and tapers to a point caudally.
2. Subrectangular.
3. D-shaped, broadest along lateral profile.

**59 - Quadrate**, shape of ventral condyle:

1. Rostrocaudally narrow and mediolaterally broad, with larger lateral condylar surface and lateral and medial condyles on same plane.
2. Asymmetrical with enlarged, more ventrally situated lateral condyle.

**60 - Squamosal**, morphology of postorbital process dorsal to *M. adductor mandibulae externus superficialis* origin site:

1. Gently convex.
2. Mediolaterally compressed and blade-like.

**61 - Squamosal**, orientation of caudomedial process (modified from Prieto-Márquez et al. 2006, character 45):

1. Curved rostromedially.
2. Curved caudomedially.
3. Straight and medially directed.

**62 - Squamosal**, relationship of right and left squamosals on skull roof (Horner et al. 2004, character 63):

1. Widely separated by parietal.
2. Separated by only a narrow band of the parietal.
3. In broad contact with each other.

**63 - Frontal**, participation in dorsal orbital rim (Norman 2002, character 19):

1. Present.
2. Absent.

**64 - Supraoccipital**, contribution to foramen magnum (You et al. 2003, character 23):

1. Present.
2. Absent, excluded by exoccipitals.

**65 - Supraoccipital**, morphology of supraoccipital-exoccipital contact (Horner et al. 2004, character 66):

1. Straight suture that meets squamosal.
2. Ventrolateral corner of supraoccipital is inset into exoccipital so that supraoccipital is locked between exoccipitals.

**66 - Supraoccipital**, inclination of caudal surface (modified from Horner et al. 2004, character 65):

1. Caudal surface rostrally inclined.
2. Caudal surface vertical.

**67 - Exoccipital-Opisthotic**, paroccipital process shape (Weishampel et al. 2003, character 13):

1. Dorsoventrally expanded distally.
2. Pendant.

**68 - Exoccipital-Opisthotic**, paroccipital process orientation of pendant distal portion (Horner et al. 2004, character 62):

1. Straight and ventrally directed.
2. Curved rostrally.

**69 - Basioccipital**, orientation of occipital condyle (modified from Prieto-Márquez 2010, character 152):

1. Caudoventrally directed.
2. Caudally directed.

**70 - Basioccipital**, rostrocaudally directed groove extending along ventral surface:

1. Absent.
2. Present.

**71 - Basioccipital**, rostrocaudally directed, sharply defined ridge between basal tubera:

1. Absent.
2. Present.

**72 - Basisphenoid**, surface between basipterygoid processes (modified from Gates & Sampson 2007, characters 78 and 79):

1. Smooth.
2. Transverse, sharply defined ridge between basipterygoid processes.
3. Ventrally directed prong between basipterygoid processes.

**73 - Basisphenoid**, orientation of basipterygoid processes (modified from Prieto-Márquez et al. 2006, character 83):

1. Ventrolaterally directed and rostrally inclined.
2. Ventrolaterally directed and caudally curved.

**74 - Foramen magnum**, composition of ventral margin (modified from Weishampel et al. 1993, character 24):

1. Caudomedial surfaces of left and right exoccipitals and dorsal margin of basioccipital.
2. Left and right exoccipitals only.

**75 - Dentary teeth**, intercrown spaces (You *et al*. 2003, character 32):

1. Present.
2. Absent.

**76 - Dentary teeth**, morphology of marginal denticles (modified from Norman 2002, character 31):

1. Tongue-shaped with smooth edges.
2. Tongue-shaped with mammillated edges.
3. Reduced to small mammillated papillae.

**77 - Dentary teeth**, number of replacement teeth per tooth position (modified from Weishampel et al. 1993, character 32):

1. One.
2. Two.
3. Three.

**78 - Dentary teeth**, number of teeth per tooth position forming part of occlusal plane (modified from Norman 2002, character 39):

1. One.
2. Two.
3. Three.

**79 - Dentary teeth**, shape of crown in lingual view (modified from Norman 2002, character 29):

1. mesiodistally broad, oblong, shield-like surface.
2. mesiodistally narrow and diamond-shaped.

**80 - Dentary teeth**, ridges on lingual surface of crown:

1. Absent.
2. Present.

**81 - Dentary teeth**, position of primary ridge (modified from You et al. 2003, character 39):

1. Mesially offset.
2. Distally offset.
3. No offset, primary ridge divides the lingual side of the crown into equal halves.

**82 - Dentary teeth**, number and morphology of ridges on lingual surface of crown:

1. Prominent primary ridge and multiple separate faint accessory ridges on both sides of it.
2. Prominent primary ridge and multiple evenly-spaced accessory ridges on either side such that entire lingual surface is corrugated.
3. Parallel and similarly prominent primary and secondary ridges with multiple faint accessory ridges arising from marginal denticles.
4. Primary ridge and a single less prominent accessory ridge on either side.
5. Primary ridge and a single mesial accessory ridge.
6. Primary ridge only.

**83 - Maxillary teeth**, intercrown spaces:

1. Present.
2. Absent.

**84 - Maxillary teeth**, number of teeth per tooth position forming part of occlusal plane:

1. One.
2. Two.

**85 - Maxillary teeth**, ridges on labial surface of crown:

1. Absent.
2. Present.

**86 - Maxillary teeth**, primary ridge position and morphology (modified from You et al. 2003, character 36):

1. Distally offset.
2. No offset, primary ridge divides the labial side of the crown into equal halves.

**87 - Maxillary teeth**, number and morphology of ridges on labial surface of crown:

1. Primary ridge with multiple parallel accessory ridges on either side.
2. Multiple ridges of similar size, such that primary ridge cannot be distinguished.
3. Primary ridge and only mesial accessory ridges.
4. Primary ridge only.

**88 - Axis**, morphology of axial neural spine in lateral view:

1. Caudodorsally sloping process.
2. Dorsally expanded process.

**89 - Cervical vertebrae**, opisthocoely of centra:

1. Slightly opisthocoelous, flat or slightly convex cranial face.
2. Deeply opisthocoelous, hemispherical cranial face protrudes beyond ventral and dorsal surfaces of centrum and has a smooth, rounded surface.

**90 - Ossified epaxial tendons**, arrangement along dorsal, sacral, and caudal vertebrae (Weishampel et al. 2003, character 42):

1. Longitudinally arranged.
2. Double-layered lattice.

**91 - Sternal**, caudolateral process (modified from Kobayashi & Azuma 2003, character 23):

1. Absent.
2. Present.

**92 - Sternal**, caudomedial process:

1. Absent.
2. Present.

**93 - Sternal**, shape of main body in dorsal or ventral view, excluding caudolateral process if present:

1. Convex medially and concave laterally.
2. Convex medially and straight laterally.

**94 - Scapula**, expansion of caudal end:

1. Gently convex expansion along caudodorsal margin, caudoventral margin tapers into hook-like flange.
2. Caudal end paddle-shaped, dorsal and ventral margins of scapula diverge towards caudal end.
3. Caudal margin of scapula straight, dorsal and ventral margins are parallel approaching caudal margin of scapula and meet caudal margin at nearly right angles.

**95 - Scapula**, acromion process orientation (Norman 2002, character 44):

1. Dorsally directed.
2. Laterally directed.

**96 - Humerus**, shape of deltopectoral crest (modified from Weishampel et al. 1993, character 37):

1. Distal margin rounded and merges gradually with the lateral margin of the humeral shaft.
2. Distal margin angular and merges abruptly with the lateral margin of the humeral shaft.

**97 - Manus**, digit I (Norman 2002, character 51):

1. Present.
2. Absent.

**98 - Manus**, ungual of digit I, shape (modified from Norman 2002, character 52):

1. Claw-like.
2. Conical or sub-conical.
3. A flattened triangle.
4. Dorsoventrally compressed conus, resembling ungual in Digit II.
5. Absent.

**99 - Manus**, arrangement of metacarpals II-IV (You et al. 2003, character 49):

1. Spreading.
2. Closely appressed.

**100 - Manus**, unguals of digits II and III, shape (Norman 2002, character 53):

1. Claw-like.
2. Flattened and hoof-like.

**101 - Manus**, digit III, number of phalanges (Weishampel et al. 2003, character 53):

1. Four.
2. Three.

**102 - Ilium**, preacetabular process, cranial end:

1. Rounded.
2. Horizontal boot.

**103 - Ilium**, preacetabular process, twisting along its length such that the dorsal surface of the ilium becomes the lateral surface of the cranial end of the process (modified from Weishampel et al. 2003, character 56):

1. Absent.
2. Present.

**104 - Ilium**, dorsal margin above pubic and ischial peduncles and acetabulum (modified from Weishampel et al. 2003, character 55):

1. Straight.
2. Convex.
3. Sinuous, convex above pubic peduncle and concave above ischial peduncle.

**105 - Ilium**, morphology of dorsal margin of postacetabular process dorsal to ischial peduncle (modified from Norman 2002, character 56):

1. Smooth surface.
2. Laterally bulging eminence dorsal to ischial peduncle, no modification of dorsal margin.
3. Mediolaterally thickened dorsal margin compared to dorsal margin above pubic peduncle.
4. Thickened and laterally-bulging everted rim along dorsal margin.
5. Laterally-projecting, non-pendant shelf continuous with dorsal margin of ilium.
6. Pendant supraacetabular process continuous with dorsal margin of ilium.

**106 - Ilium**, postacetabular process, shape in lateral view (modified from Norman 2002, character 57):

1. Rounded with break in slope along dorsal margin, parallel dorsal and ventral margins.
2. Tapers to a point with break in slope along dorsal margin, forming a distinct platform for the origin of *M. iliocaudalis*.
3. Tapers with no break in slope along dorsal margin.
4. Subrectangular with no break in slope.

**107 - Ilium**, postacetabular process, promontory in their dorsal margin:

1. Absent.
2. Present, the dorsal margin arises abruptly at the supracetabular region.

**108 - Pubis**, cranial expansion of cranial pubic process (modified from Norman 2002, character 58):

1. Absent, dorsal and ventral margins parallel or nearly parallel.
2. Present, dorsal and ventral margins diverge distally.

**109 - Pubis**, dorsal margin:

1. Straight.
2. Concave.

**110 - Pubis**, caudal pubic process (Norman 2002, character 59):

1. Approximately equal in length to ischium.
2. Shorter than ischium.

**111 - Pubis**, caudal pubic process, morphology of distal end:

1. Rounded.
2. Tapers to a point.

**112 - Ischium**, morphology of shaft (modified from Norman 2002, character 60):

1. Curved caudally.
2. Curved cranially.
3. Straight.

**113 - Ischium**, morphology of distal end (modified from Prieto-Márquez et al. 2006, character 126):

1. The shaft becomes more or less wider, no cranial expansion is present.
2. Cranially expanded.
3. Bluntly truncated.

**114 - Femur**, curvature of shaft in lateral view (Norman 2002, character 62):

1. Distal half of shaft curved caudally.
2. Distal half of shaft straight.

**115 - Femur**, groove on caudal aspect of femoral head (Winkler et al. 1997, character 25):

1. Present.
2. absent.

**116 - Femur**, morphology of fourth trochanter (Norman 2002, character 63):

1. Pendant.
2. Broad and triangular.
3. Curved, laterally compressed eminence.

**117 - Femur**, location of fourth trochanter (Weishampel et al. 2003, character 69):

1. Arises on proximal half of femoral shaft.
2. Arises at midshaft of femur.

**118 - Femur**, location of insertion scar of *M. caudifemoralis longus* (Ruiz-Omeñaca et al. 2006):

1. extends from fourth trochanter onto medial surface of femoral shaft.
2. widely separated from fourth trochanter, restricted to medial surface of femoral shaft.

**119 - Femur**, intercondylar extensor groove (Weishampel et al. 2003, character 70):

1. Absent.
2. Present.

**120 - Femur**, intercondylar extensor groove (modified from Norman 2002, character 64; McDonald *et al*. 2010, character 127; Barrett *et al*. 2011, character 127):

1. Broad, shallow, V-shaped, edges of groove meet at greater than 90 degrees to one another.
2. Tight, deep, V-shaped, edges of groove meet at less than 90 degrees.
3. Deep, narrow, U-shaped, partially enclosed by slight expansion of medial condyle.
4. Deep, U-shaped, partially enclosed by expansion of medial and lateral condyles.
5. Canal fully enclosed by fusion of lateral and medial condyles.

**121 - Femur**, intercondylar flexor groove (modified from Weishampel et al. 2003, character 71):

1. Completely open, U-shaped trough.
2. Partially closed by lateral inflation of medial condyle.

**122 - Pes**, prominent lip extending proximodorsally from dorsal margins of phalangeal proximal articulation facets:

1. Present.
2. Absent.

**123 - Pes**, morphology of unguals on digits II-IV (modified from Norman 2002, character 67):

1. Dorsoventrally flattened, but elongate and pointed.
2. Dorsoventrally flattened and elongate, but with blunt truncated tips.
3. Hoof-like shape.

**124 - Premaxilla**, transverse ridge of thickened bone caudal to oral margin, separated from the oral margin by a deep sulcus bearing vascular foramina (modified from Prieto-Márquez 2010, character 63):

1. Absent.
2. Present.

**125 - Ilium**, brevis fossa, transverse width (from Barrett *et al*. 2011, character 132):

1. Narrow.
2. Very broad and expanding in width towards its caudal margin such that it appears triangular in dorsal or ventral view.

**126 - Femur**, deep cleft separating the greater and lesser trochanters (modified from Barrett *et al*. 2011, character 134):

1. Present.
2. Absent, lesser trochanter is closely appressed to the proximal end of the femur.

**127 - Metatarsals III and IV,** deep caudolateral notch on MT III for the reception of a prominent process of MT IV (modified from Barrett *et al*. 2011, character 135):

1. Absent.
2. Present.

**128 - Dentary**, general profile of the dorsal margin of the dentary rostral region which articulates with the predentary in medial or lateral view (modified from Prieto-Márquez & Wagner 2009, character 45):

1. A relatively steep dorsal margin, forming a prominent depression in the dentary symphyseal region.
2. A smooth and gradually descending dorsal margin in the rostrodorsal region of the dentary.

**129 - Dentary**, rostral tip raised respect to the ventral margin:

1. Present.
2. Absent.

**130 - Maxilla**, location of line of alveolar ("special") foramina on medial side (modified from Sues & Averinov 2009 character 55):

1. Located ventral to mid-height of maxilla.
2. Roughly at the mid-height.
3. Located dorsal to mid-height of maxilla.

**131 - Palpebral bone** (from Norman 2002, character 13):

1. Present.
2. Absent or fused to orbital margin.

**132 - Quadrate**, position of the quadratojugal notch relative to the dorsoventral height of the quadrate (modified from Prieto-Márquez & Wagner 2009, character 125):

1. The midpoint of the quadratojugal notch is located near the half dorsoventral height of the quadrate.
2. The midpoint of the quadratojugal notch is located ventral to the midpoint of the quadrate height.

**133 - Quadrate**, buttress just below the dorsal condyle of the quadrate:

1. Absent or poorly developed as a gentle convexity.
2. Present, the buttress is a sharp and hanging protuberance.
3. Present, the buttress is vertical along the caudodorsal margin of the quadrate.

**134 - Skull**, transverse width of the cranium in postorbital region in dorsal view (from Sues & Averinov 2009, character 73):

1. Broad, width maintained from orbit to quadrate head.
2. Distinctly narrowed at level of quadrate heads.

**135 - Sacral vertebrae**, number (only truly sacrals) (modified from Sues & Averinov 2009, character 105):

1. 5.
2. 6.
3. 7 or more.

**136 - Coracoid**, morphological character of the hook-shaped ventral process of the coracoid (corresponding with Horner et al. 2004, character 79):

1. Relatively short and directed ventrally.
2. Relatively long, recurved and directed caudoventrally.

**137 - Coracoid**, foramen on its external surface:

1. Present.
2. Absent, a notch opens between the glenoid and the scapular process.

**138 - Humerus**, deltopectoral crest length (from Sues & Averinov 2009, character 112):

1. Short, much less than half length of humerus.
2. Extends at least to midshaft or longer.

**139 – Manus,** metacarpal I shape (from Norman 2002, character 49)**:**

1. Normal and free elongate bone.
2. Short, block-like set against carpals.
3. Absent.

**140 - Manus**, phalanx 1of the digit I:

1. Large.
2. Reduced to a flattened disc.
3. Absent.

**141 – Pubis,** craniocaudal length of the cranial blade of the prepubic process relative to that of the caudal neck (caudal constriction) of the prepubic process (from Xing et al. 2014, character 331):

1. The length of the cranial blade is less than that of the caudal neck.
2. The length of the cranial blade is equal to or greater than that of the caudal neck.

**142 - Pubis**, orientation of the dorsoventral expansion of the prepubic process (modified from Prieto-Márquez 2010, character 252):

1. Equal expansion of both dorsal and ventral margin in the most cranial region of the preacetabular blade, no orientation is adopted, cranially directed.
2. The dorsal region of the expansion is more expanded than the ventral region, so that distally the process is dorsally directed.
3. The ventral region is more expanded than the dorsal region, so that the distal expansion is ventrally directed.

**143 - Ischium**, orientation of the craniocaudal axis of the ischial pubic peduncle relative to the ischial shaft in parallel with the level (modified from Xing et al. 2014, character 322):

1. Directed cranioventrally, non-aligned with the ischial shaft.
2. Almost directed cranially, aligned with ischial shaft.

**144 - Femur**, femoral shaft bowed laterally:

1. Absent.
2. Present.

**145 - Femur**, cranial expansion of distal end:

1. Absent.
2. Present, in medial views the distal end projects cranially more than femoral shaft does.

**146 - Femur**, caudal expansion of both condyles:

1. Lateral and medial condyles have similar caudal expansion.
2. Medial condyle is larger than lateral one.

**147 – Pes,** metatarsal I (from Norman 2002, character 66):

1. Well-developed and articulates with proximal phalanx.
2. Slender, splint-like.
3. Absent.

**References**

Barrett PM, Butler RJ, Twitchett RJ, Hutt S. New material of *Valdosaurus canaliculatus* (Ornithischia: Ornithopoda) from the Lower Cretaceous of Southern England. Special Papers in Palaeontology. 2011; 86:131–163.

Gates TA, Sampson SD. A new species of *Gryposaurus* (Dinosauria: Hadrosauridae) from the late Campanian Kaiparowits Formation, southern Utah, USA. Zoological Journal of the Linnean Society. 2007; 151:351–376.

Head JJ. A new species of basal hadrosaurid (Dinosauria, Ornithischia) from the Cenomanian of Texas. Journal of Vertebrate Paleontology. 1998; 18:718–738.

Horner JR, Weishampel DB, Forster CA. The Dinosauria: Second Edition. University of California Press: Berkeley. Chapter 20, Hadrosauridae. 2004; p. 438–463.

Kobayashi Y, Azuma Y. A new iguanodontian (Dinosauria: Ornithopoda) from the Lower Cretaceous Kitadani Formation of Fukui Prefecture, Japan. Journal of Vertebrate Paleontology. 2003; 23:166–175.

McDonald AT, Wolfe DG & Kirkland JI. A new basal hadrosauroid (Dinosauria: Ornithopoda) from the Turonian of New Mexico. Journal of Vertebrate Paleontology. 2010; 30:799–812.

Norman DB. On Asian ornithopods (Dinosauria: Ornithischia). 4. *Probactrosaurus* Rozhdestvensky, 1966. Zoological Journal of the Linnean Society. 2002; 136:113–144.

Norman DB. The Dinosauria: Second Edition. University of California Press: Berkeley. Chapter 18, Basal Iguanodontia; 2004; p. 413–437.

Prieto-Márquez A. Global phylogeny of hadrosauridae (Dinosauria: Ornithopoda) using parsimony and Bayesian methods. Zoological Journal of the Linnean Society. 2010; 159:435–502.

Prieto-Márquez A, Wagner JR. *Pararhabdodon isonensis* and *Tsintaosaurus spinorhinus*: a new clade of lambeosaurine hadrosaurids from Eurasia. Cretaceous Research. 2009; 30:1238–1246.

Prieto-Márquez A, Gaete R, Rivas G, Galobart À, Boada M. Hadrosauroid dinosaurs from the Late Cretaceous of Spain: *Pararhabdodon isonensis* revisited and *Koutalisaurus kohlerorum*, gen. et sp. nov. Journal of Vertebrate Paleontology. 2006; 26:929–943.

Ruiz-Omeñaca JI, Pereda Suberbiola X, Galton PM. Horns and Beaks: Ceratopsian and Ornithopod Dinosaurs. Indiana University Press: Bloomington. Chapter 1, *Callovosaurus leedsi*, the earliest dryosaurid dinosaur (Ornithischia: Euornithopoda) from the Middle Jurassic of England; 2006; p. 3–16.

Sues HD, Averianov A. A new basal hadrosauroid dinosaur from the Late Cretaceous of Uzbekistan and the early radiation of duck–billed dinosaurs. Proceedings of The Royal Society of London B. 2009; 276:2549–2555.

Verdú FJ, Royo-Torres R, Cobos A, Alcalá L. New systematic and phylogenetic data about the early Barremian *Iguanodon galvensis* (Ornithopoda: Iguanodontoidea) from Spain. Historical Biology. 2018; 30(4):437–474.

Weishampel DB, Norman DB, Grigorescu D. *Telmatosaurus transsylvanicus* from the Late Cretaceous of Romania: the most basal hadrosaurid dinosaur. Palaeontology. 1993; 36:361–385.

Weishampel DB, Jianu C-M, Cziki Z, Norman DB. Osteology and phylogeny of *Zalmoxes* (n. g.), an unusual ornithopod dinosaur from the latest Cretaceous of Romania. Journal of Systematic Palaeontology. 2003; 1:123-143.

Winkler DA, Murry PA, Jacobs LL. A new species of *Tenontosaurus* (Dinosauria: Ornithopoda) from the Early Cretaceous of Texas. Journal of Vertebrate Paleontology. 1997; 17:330–348.

Xing H, Wang D, Han F, Sullivan C, Ma Q, He Y, Hone DWE, Yan R, Du F, Xu X. A New Basal Hadrosauroid Dinosaur (Dinosauria: Ornithopoda) with Transitional Features from the Late Cretaceous of Henan Province, China. PLoS ONE. 2014; 9: e98821.

You H, Luo Z, Shubin NH, Witmer LM, Tang Z, Tang F. The earliest-known duck-billed dinosaur from deposits of late Early Cretaceous age in northwest China and hadrosaur evolution. Cretaceous Research. 2003; 24:347–355.
