# Supplementary material for: A new styracosternan hadrosauroid (Dinosauria: Ornithischia) from the Early Cretaceous of Portell, Spain
Source: PLoS One. 2021 Jul 7;16(7):e0253599. doi: 10.1371/journal.pone.0253599 (PMC8262792; doi:10.1371/journal.pone.0253599)
Supplement: S5 File — (DOCX) [file pone.0253599.s005.docx]

**Supporting information 3 - Data Set Modifications**

We realized a phylogenetic analysis using original characters from Verdú et al. (2018). We revised the data set in its matrix and we added some missing jaw characters and modified some others.

Below we present the two taxa added to the data set from Verdú et al. (2018) and the characters modified or revised. List of characters used are in the Supporting information 2 (S2_File).

Institutional abbreviations and references can be found at the end of this document.

**Summary table**

| **Added taxa** | *Magnamanus soriaensis* |
| --- | --- |
|  | *Portellsaurus sosbaynati* gen. et sp. nov. |

| **Taxa** | **Changed characters** | **Based on** | **Comments** | **References** |
| --- | --- | --- | --- | --- |
| *Barilium dawsoni* | 14 (from ? to 0) | NHMUK PV 28660 in Fig. 2 in McDonald et al. (2010). | *Kukufeldia tilgatensis* is formerly considered to be junior synonym of *Barilium dawsoni* (Norman [2015]). Changes based on the specimen NHMUK PV 28660 and McDonald's (2012a) data set. | McDonald et al. 2010; Norman 2011 |
|  | 19 (from ? to 0) | NHMUK PV 28660 in Fig. 2 in McDonald et al. (2010). |  |  |
| *Bolong yixianensis* | 14 (from ? to 1) | YHZ-001 (holotype) in Fig. 19.3 in Wu and Godefroit 2012. |  | Wu and Godefroit 2012 |
| *Hypselospinus* *fittoni* | 14 (from ? to 2) | NHMUK PV R 1831 in McDonald's (2012a) data set. | Return to McDonald's (2012a) original character state | Norman 2015 |
|  | 15 (from ? to 0) | NHMUK PV R 1831 in Fig. 36 in Norman (2015). | The dentary NHMUK PV R 1831 is referable to *Hypselospinus fittoni* according to Norman (2015). |  |
|  | 19 (from ? to 0) | NHMUK PV R 1831 in Fig. 36 in Norman (2015). |  |  |
|  | 20 (from ? to 0) | NHMUK PV R 1831 in Fig. 36 in Norman (2015). |  |  |
|  | 21 (from ? to 1) | NHMUK PV R 1831 in Fig. 36 in Norman (2015). |  |  |
|  | 22 (from ? to 0) | NHMUK PV R 1831 in Fig. 36 in Norman (2015). |  |  |
|  | 23 (from ? to 0) | NHMUK PV R 1831 in Fig. 36 in Norman (2015). |  |  |
|  | 128 (from 0 to 1) | NHMUK PV R 1831 in Fig. 36 in Norman (2015). |  |  |
| *Lanzhousaurus magnidens* | 21 (from 1 to 0) | FRDC: GSLTZP 1-1（holotype) in Fig. 1 A, D in You et al. (2005). | Return to You et al. (2005) original character state | You et al. 2005 |
| *Mantellisaurus atherfieldensis* | 21 (from 1 to 0) | Fig. 19 in Norman (1986). | We consider *Dollodon bampingi* (RBINS R57) to be a junior synonym of *Mantellisaurus atherfieldensis* following McDonald (2012b) and Norman (2012). | Hooley 1925; Norman 1986; McDonald 2012b |
|  | 22 (from 0 to ?) | Fig. 19 in Norman (1986). |  |  |
|  | 23 (from 0 to ?) | Fig. 19 in Norman (1986). |  |  |
| *Ouranosaurus nigeriensis* | 14 (from 2 to 1) | MNHN GDF 300 (holotype) in Fig. 29 and Plate XIX.5 in Taquet (1976). |  | Taquet 1976 |
|  | 21 (from 1 to 0), | MNHN GDF 300 (holotype) in Fig. 29, p.95 and Plate XIX.5 in Taquet (1976). |  |  |
|  | 22 (from 1 to ?), | MNHN GDF 300 (holotype) in Fig. 29, p.95 and Plate XIX.5 in Taquet (1976). | Return to Taquet (1976) original character state |  |
|  | 23 (from 0 to ?) | MNHN GDF 300 (holotype) in Fig. 29, p.95 and Plate XIX.5 in Taquet (1976). | Return to Taquet (1976) original character state |  |
| *Owenodon hoggii* | 10 (from 1 to 2) | NHMUK PV R 2998 (holotype) in Fig. 2 in Norman and Barrett 2002. |  | Norman and Barrett 2002 |
| *Proa valdearinnoensis* | 19 (from 1 to 0) | AR-1-1365, AR-1-1366 (paratype) in Fig. 6 in McDonald et al. 2012b. |  | McDonald et al. 2012b |

**Institutional abbreviations**

**FRDC**- *Fossil Research and Development Center of the Third Geology and Mineral Resources Exploration Academy of Gansu Province*, Lanzhou, Gansu Province, China; **MNHN** - *Musée national d’Histoire naturelle*, Paris, France; **NHMUK -** *Natural History Museum*, London, UK; **RBINS –** *Royal Belgian Institute of Natural Sciences*, Brussels, Belgium.

**References**

Hooley RW. On the skeleton of *Iguanodon atherfieldensis* sp. nov., from the Wealden Shales of Atherfield (Isle of Wight). Quarterly Journal of the Geological Society of London. 1925; 81: 1–61.

McDonald AT, Barrett PM, Chapman SD. A new basal iguanodontian (Dinosauria: Ornithischia) from the Wealden (Lower Cretaceous) of England. Zootaxa. 2010; 2569: 1–43.

McDonald AT. Phylogeny of basal iguanodonts (Dinosauria: Ornithischia): an update. PLoS ONE. 2012a; 7: e36745.

McDonald AT. The status of *Dollodon* and other basal iguanodonts (Dinosauria: Ornithischia) from the Lower Cretaceous of Europe. Cretaceous Research. 2012b; 33: 1–6.

Norman DB. On the anatomy of *Iguanodon atherfieldensis* (Ornithischia: Ornithopoda). Bulletin de l'Institut Royal des Sciences Naturelles de Belgique. 1986; 56: 281–372.

Norman DB. On the osteology of the lower Wealden (Valanginian) iguanodontian *Barilium dawsoni* (Iguanodontia: Styracosterna). Special Papers in Palaeontology. 2011; 86: 1–28.

Norman DB. Bernissart Dinosaurs and Early Cretaceous Terrestrial Ecosystems. Indiana University Press: Bloomington. Chapter 15, Iguanodontian Taxa (Dinosauria: Ornithischia) from the Lower Cretaceous of England and Belgium. 2012; p. 174–212.

Norman DB. On the history, osteology and systematic position of the Wealden (Hastings group) dinosaur *Hypselospinus fittoni* (Iguanodontia: Styracosterna). Zoological Journal of the Linnean Society. 2015; 173: 92–189.

Norman DB, Barrett PM. Ornithischian dinosaurs from the Lower Cretaceous (Berriasian) of England. Special Papers in Palaeontology. 2002; 68: 161–189.

Taquet P. Géologie et Paléontologie du Gisement de Gadoufaoua (Aptien du Niger) [Geology and palaeontology of the Gadoufaoua locality (Aptian of Niger)]. Editions du Centre National de la Recherche Scientifique: París.Chapter 3, Ostéologie d’Ouranosaurus nigeriensis, Iguanodontide du Crétacé Inférieur du Niger [Osteology of Ouranosaurus nigeriensis, Lower Cretaceous Iguanodontid from Niger]. 1976; p. 57–168.

Verdú FJ, Royo-Torres R, Cobos A, Alcalá L. New systematic and phylogenetic data about the early Barremian *Iguanodon galvensis* (Ornithopoda: Iguanodontoidea) from Spain. Historical Biology. 2018; 30(4):437–474.

Wu W, Godefroit P. Anatomy and relationships of *Bolong yixianensis*, an Early Cretaceous iguanodontoid dinosaur from western Liaoning, China. In: Godefroit P, editor. Bernissart Dinosaurs and Early Cretaceous Terrestrial Ecosystems. Bloomington: Indiana University Press; 2012. pp. 292–333.

You H-L, Ji Q, Li D-Q. *Lanzhousaurus magnidens* gen. et sp. nov from Gansu Province, China: the largest-toothed herbivorous dinosaur in the world. Geological Bulletin of China. 2005; 24: 785–794.
